# BCR-ABL Tyrosine Kinase Inhibitors: Which Mechanism(s) May Explain the Risk of Thrombosis?

**DOI:** 10.1055/s-0038-1624566

**Published:** 2018-02-14

**Authors:** Hélène Haguet, Jonathan Douxfils, Christian Chatelain, Carlos Graux, François Mullier, Jean-Michel Dogné

**Affiliations:** 1 University of Namur, Namur Thrombosis and Hemostasis Center (NTHC), Namur Research Institute for Life Sciences (NARILIS), Department of Pharmacy, Namur, Belgium; 2 Université catholique de Louvain, CHU UCL Namur, Namur Thrombosis and Hemostasis Center, Hematology Laboratory, Yvoir, Belgium; 3 Université catholique de Louvain, CHU UCL Namur, Namur Thrombosis and Hemostasis Center, Department of Hematology, Yvoir, Belgium; 4 QUALIblood s.a., Namur, Belgium

**Keywords:** BCR-ABL, arterial thrombotic events, tyrosine kinase inhibitors, chronic myeloid leukemia

## Abstract

Imatinib, the first-in-class BCR-ABL tyrosine kinase inhibitor (TKI), had been a revolution for the treatment of chronic myeloid leukemia (CML) and had greatly enhanced patient survival. Second- (dasatinib, nilotinib, and bosutinib) and third-generation (ponatinib) TKIs have been developed to be effective against BCR-ABL mutations making imatinib less effective. However, these treatments have been associated with arterial occlusive events. This review gathers clinical data and experiments about the pathophysiology of these arterial occlusive events with BCR-ABL TKIs. Imatinib is associated with very low rates of thrombosis, suggesting a potentially protecting cardiovascular effect of this treatment in patients with BCR-ABL CML. This protective effect might be mediated by decreased platelet secretion and activation, decreased leukocyte recruitment, and anti-inflammatory or antifibrotic effects. Clinical data have guided mechanistic studies toward alteration of platelet functions and atherosclerosis development, which might be secondary to metabolism impairment. Dasatinib, nilotinib, and ponatinib affect endothelial cells and might induce atherogenesis through increased vascular permeability. Nilotinib also impairs platelet functions and induces hyperglycemia and dyslipidemia that might contribute to atherosclerosis development. Description of the pathophysiology of arterial thrombotic events is necessary to implement risk minimization strategies.

## Introduction

In 2001, the approval of *imatinib* , the first-in-class tyrosine kinase inhibitor (TKI) targeting BCR-ABL, transformed the prognosis of patients with chronic-phase (CP) chronic myeloid leukemia (CML) from a life-threatening condition to a manageable and chronic disease. 1 Yet, despite satisfactory outcomes, 33% of patients did not achieved optimal response because of treatment resistance or intolerance. 1 The identification of the predominant resistance mechanism (i.e., point mutations in the kinase domain of Bcr-Abl) led to the development of second-generation BCR-ABL TKIs (dasatinib, nilotinib, and bosutinib, respectively, approved in 2006, 2007, and 2012) active against most of the BCR-ABL mutated forms. 2 3 Second-generation TKIs demonstrated no or little improvement of the overall survival compared with imatinib, 4 5 6 but two of these (i.e., dasatinib and nilotinib) improve surrogate outcomes and permit quicker and deeper achievement of molecular response, which is criteria to try treatment cessation (i.e., MR 4 or higher molecular response stable for at least 2 years). 7 Based on these results, dasatinib and nilotinib were approved in 2010 for frontline management of CML, whereas bosutinib is used only after failure or intolerance of first-line BCR-ABL TKIs. Unfortunately, these treatments were ineffective against a common mutation (14% of all mutations) in the gatekeeper residue of BCR-ABL (i.e., the T315I a mutation), 8 9 10 requiring the development of a third-generation TKI (ponatinib), efficient against this mutation. Ponatinib is currently the only treatment active against the T315I mutation and is therefore reserved for patients with this mutation or for patients resistant to frontline treatments. 11

Since its approval, the first-generation TKI, imatinib, has demonstrated reassuring safety profile, with low rate of grade 3/4 adverse events and excellent tolerability. 12 13 Conversely, new-generation BCR-ABL TKIs—nilotinib, dasatinib, bosutinib, and ponatinib—are more recent and display different safety profile. Dasatinib, nilotinib, and ponatinib are largely associated with fluid retention and dasatinib specifically induces high rate of pleural effusions. 14 15 16 17 18 Nilotinib induces metabolic disorders such as dyslipidemia and hyperglycemia, whereas bosutinib safety profile is mainly characterized by gastrointestinal events (i.e., diarrhea, nausea, vomiting). 19 20 Finally, ponatinib has been rapidly associated with high rate of vascular occlusion. 21

Recently, meta-analyses of randomized clinical trials established that ponatinib is not the only new-generation TKI that increases the cardiovascular risk. 22 23 The four new-generation BCR-ABL TKIs increase the risk of vascular occlusive events compared with imatinib, especially arterial occlusive diseases, and this is in accordance with clinical trial data. 22 23 24 25 However, this cardiovascular risk is controversy for dasatinib because of the low incidence (1.1 per 100 patient-year) of cardiovascular events in clinical trials. 26 27 Recently, a large retrospective analysis of CP-CML patients treated with BCR-ABL TKIs at the *MD Anderson Cancer Center* confirmed the increased risk of vascular occlusive events with dasatinib. 28 Another controversial point is the effect of imatinib on the cardiovascular system. Indeed, imatinib is associated with low risk of cardiovascular events and it was therefore hypothesized that imatinib prevents their occurrence. 29 30 Clinical data indicate that most patients developing arterial occlusive events with new-generation BCR-ABL TKIs are high-risk patients, but cardiovascular events also occurred in young and healthy patients. Additional information on clinical safety of BCR-ABL TKIs is described in the Supplementary Material ( Table S1 ). We assume that the mechanism underlying arterial thrombosis with BCR-ABL TKIs might be multiple. The predominance of arterial events raised concerns about the impact of BCR-ABL TKIs on platelet functions, atherosclerosis, and metabolism, and precluded prothrombotic states to be responsible of these events. 31

This review particularly focuses on the contribution of glucose and lipid metabolism, atherosclerosis, and platelets in the occurrence of cardiovascular events with new-generation TKIs. The last section discusses relevant off-targets that might be implicated in the cardiovascular toxicity. The discovery of the mechanism(s) by which arterial occlusive events arose in CML patients would help in the management of patients treated with BCR-ABL TKIs and implement risk minimization measures. Discovery of the pathophysiology of these events in CML patients might also led to the development of predictive biomarkers or to the development of new therapies with no or reduced cardiovascular toxicity profile while keeping an unaltered efficacy.

## Impact on Platelet Functions

**Fig. 1:**
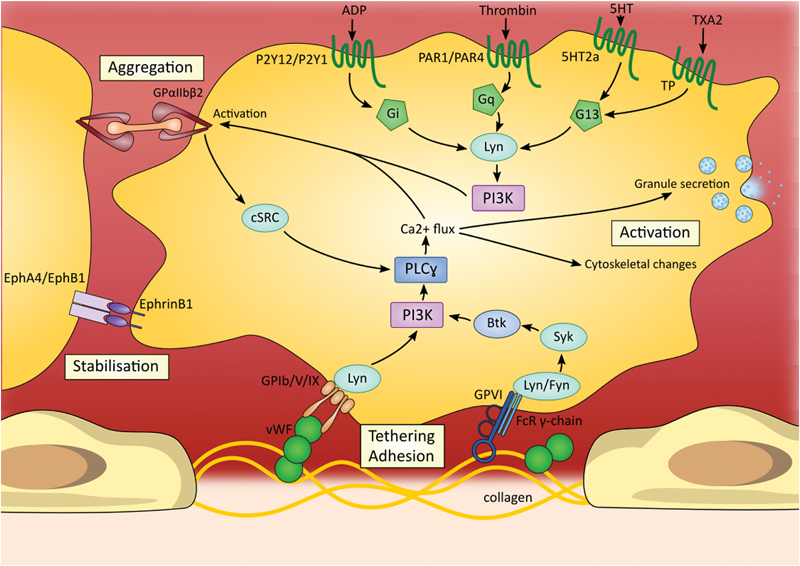
Signaling pathways supporting platelet adhesion, activation, and aggregation. Tyrosine kinases are involved in several pathways and contribute to platelet adhesion, aggregation, and activation. Important players in platelet signaling are members of the Src family kinases; particularly Lyn, Fyn, and cSRC. These three tyrosine kinases are inhibited by dasatinib which might explain platelet dysfunction encountered with this treatment. Additionally, dasatinib also inhibits BTK, Syk, EphA4, and EphB1—four tyrosine kinases involved in platelet activation and aggregate stabilization. 5HT, 5-hydroxytryptamine; ADP, adenosine diphosphate; Btk, Bruton's tyrosine kinase; Ca, calcium; Eph, ephrin; FcR, Fc receptor; GP, glycoprotein; PAR, protease-activated receptor; PI3K, phosphoinositide 3-kinase; PLC, phospholipase C; TXA2, thromboxane A2; vWF, Von Willebrand factor.

BCR-ABL TKIs are associated with both bleeding and thrombotic complications. Table 1 describes experiments assessing the impact of BCR-ABL TKIs on platelet production and functions. Imatinib and dasatinib induce hemorrhagic events in patients with CML. Interestingly, dasatinib-associated hemorrhages occurred both in patients with and without thrombocytopenia. 32 In vitro and in vivo investigations demonstrated that dasatinib affects both platelet functions (i.e., platelet aggregation, secretion, and activation) and platelet formation by impairment of megakaryocyte migration. 33 34 35 36 Furthermore, dasatinib decreases thrombus formation in vitro, in vivo, and ex vivo, 34 and decreases the number of procoagulant platelets (i.e., phosphatidylserine-exposing platelets). 35 Several dasatinib off-targets are implicated in platelet signaling and functions including members of the SFKs (e.g., Src, Lyn, Fyn, Lck, and Yes) ( Fig. 1 ). 37 38 However, SFKs are also inhibited by bosutinib without disturbance of platelet aggregation and adhesion. Dasatinib also inhibits Syk, BTK, and members of the ephrin family b (e.g., EphA2), all known to be involved in platelet functions.

**Table 1:** In vitro and ex vivo investigations of the effects of BCR-ABL TKIs on platelet production and functions

Endpoints	Methods	Models	TKIs	Findings	Ref.
Platelet production	Platelet count	Murine whole blood	Dasatinib	Thrombocytopenia platelet production	33
	Flow cytometry (DNA ploidy) Migration assay (Dunn chamber)	Megakaryocyte primary culture	Dasatinib	megakaryocyte differentiation megakaryocyte migration proplatelet formation	33
Platelet aggregation	Born aggregometry; Light transmission aggregometry	Washed human platelet	Imatinib	= CRP-, collagen- and thrombin-induced platelet aggregation	38 39 42
	Light transmission aggregometry	Human platelet (PRP)	Imatinib	ADP-induced platelet aggregation collagen- and CRP-induced platelet aggregation	34
	Light transmission aggregometry, immunostaining (PAC-1)	Human platelet (PRP); patient blood	Dasatinib	ADP-, collagen-, thrombin- and CRP-induced platelet aggregation	34 35 38
	Light transmission aggregometry; Born aggregometry	Human platelet (PRP); Washed human platelet	Nilotinib	= platelet aggregation	34 39 42
	Born aggregometry	Washed human platelet	Ponatinib	CRP-induced platelet aggregation = thrombin-induced platelet aggregation	42
Platelet activation	Immunostaining (PS)	Washed human platelet	Imatinib	= PS exposure	42
	Western blot	Human platelet lysate	Imatinib	= Src, Lyn, LAT, and BTK activation	42
	Immunostaining (PS)	Patient blood	Dasatinib	PS exposure	35
	Immunostaining (PS)	Washed human platelet	Nilotinib	= PS exposure	42
	Immunostaining (PS)	Patient blood	Nilotinib	PS exposure	35
	Western blot	Human platelet lysate	Nilotinib	= Src, Lyn, LAT and BTK activation	42
	Immunostaining (PS)	Patient blood	Bosutinib	PS exposure	35
	Immunostaining (PS)	Washed human platelet, patient blood	Ponatinib	PS exposure	35 42
	Western blot	Human platelet lysate	Ponatinib	Src, Lyn, LAT and BTK activation	42
Granule release	Immunostaining (P-selectin)	Human platelet	Imatinib	thrombin-, PAR-1- and CRP-mediated α-granule release = PAR-4-mediated α-granule release	34
	Immunostaining (P-selectin)	Washed human platelet	Imatinib	= α-granule release	42
	Immunostaining (P-selectin)	Human platelet	Dasatinib	thrombin-, PAR-1-, PAR-4- and CRP-mediated α-granule release	34
	Immunostaining (P-selectin)	Washed human platelet	Nilotinib	= PAR-4-, CRP- and thrombin-mediated α-granule release	34 42
	Immunostaining (P-selectin)	Murine platelet	Nilotinib	CRP-, PAR-4- and thrombin-mediated α-granule release	34
	Immunostaining (P-selectin)	Human platelet	Nilotinib	PAR-1-mediated α-granule release	34
	Immunostaining (P-selectin)	Washed human platelet	Ponatinib	α-granule release	42
Platelet spreading	Microscopy (platelet spreading)	Washed human platelet	Imatinib	= platelet spreading and lamellipodia formation	42
	Microscopy (platelet spreading)	Washed human platelet	Nilotinib	= platelet spreading and lamellipodia formation	42
	Microscopy (platelet spreading)	Washed human platelet	Ponatinib	platelet spreading and lamellipodia formation	42
Thrombus formation	In vitro flow study, PFA-100	Human blood, murine whole blood	Imatinib	= platelet deposition and thrombus volume = closure time	34 36 44
	Ex vivo and in vitro flow study	Murine whole blood, human whole blood	Imatinib	thrombus volume and aggregate formation	34 42
	In vitro and ex vivo flow study	Human blood, murine whole blood, patient whole blood	Dasatinib	thrombus volume and platelet deposition	34 35 36
	PFA-100	Human whole blood	Dasatinib	closure time (collagen/epinephrine activation) = closure time (collagen/ADP activation)	44
	Ex vivo flow study	Murine whole blood, patient whole blood	Nilotinib	thrombus volume (growth and stability)	34
	In vitro flow study	Human whole blood, murine whole blood	Nilotinib	= platelet deposition and thrombus volume	34 36 42
	In vitro flow study	Human blood	Bosutinib	platelet deposition (late)	36
	PFA-100	Patient blood	Ponatinib	closure time	41
	In vitro flow study	Human whole blood	Ponatinib	aggregate formation	42

Abbreviations: ADP, adenosine diphosphate; BTK, Bruton's tyrosine kinase; CRP, C-reactive protein; DNA, deoxyribonucleic acid; LAT, linker for activation of T-cells; PAR, protease-activated receptor; PFA, platelet function assay; PRP, platelet-rich plasma; PS, phosphatidyl serine.

Experimental assessments of platelet functions with imatinib demonstrate less pronounced effects on platelets. Imatinib inhibits platelet aggregation only at high doses, 34 and does not interfere with platelet aggregation in vivo. 39 However, in vitro studies also indicate decreased platelet secretion and activation by imatinib. 34 The mechanism by which imatinib inhibits platelet functions is unknown. Oppositely to dasatinib, imatinib does not inhibit SFKs, ephrins, BTK, and Syk. A hypothesis also suggests that imatinib induces bleeding disorders because of BCR-ABL rearrangements in megakaryocytic cell lines, leading to clonal expansion of dysfunctional megakaryocytes. 40

Even if ponatinib induces very few bleeding disorders, assessment of primary hemostasis in CML patients demonstrated that ponatinib induces defect in platelet aggregation. This impairment was found at all ponatinib dosage, in patients with or without low platelet counts. 41 These results were in accordance with in vitro studies which previously demonstrated similar characteristics than dasatinib (i.e., decrease of platelet spreading, aggregation, P-selectin secretion, and phosphatidylserine exposure). 35 42 However, in vitro assays tested ponatinib at 1 µM, a dose far higher than the concentration observed in patients on treatment. 43 Nilotinib and bosutinib are not associated with bleeding disorders in CML patients. First in vitro studies demonstrated little or no effect on platelet aggregation and activation with these two TKIs. 36 39 44 However, recent experiments described prothrombotic phenotype of platelets induced by nilotinib, with increase of PAR-1 c –mediated platelet secretion, adhesion, and activation, without disturbing platelet aggregation. 34 Additional studies demonstrated that nilotinib increases secretion of adhesive molecules as well as thrombus formation and stability ex vivo. 34

To summarize, dasatinib and imatinib induce hemorrhagic events through alteration of platelet functions, but the molecular mechanism needs to be better determined. Ponatinib also impairs platelet functions. Therefore, no current data involve platelets in the pathogenesis of arterial thrombosis occurring with dasatinib and ponatinib. Oppositely, nilotinib might induce arterial thrombosis through alteration of platelet secretion, adhesion, and activation.

## Metabolic Dysregulation

### Glucose Metabolism

**Fig. 2:**
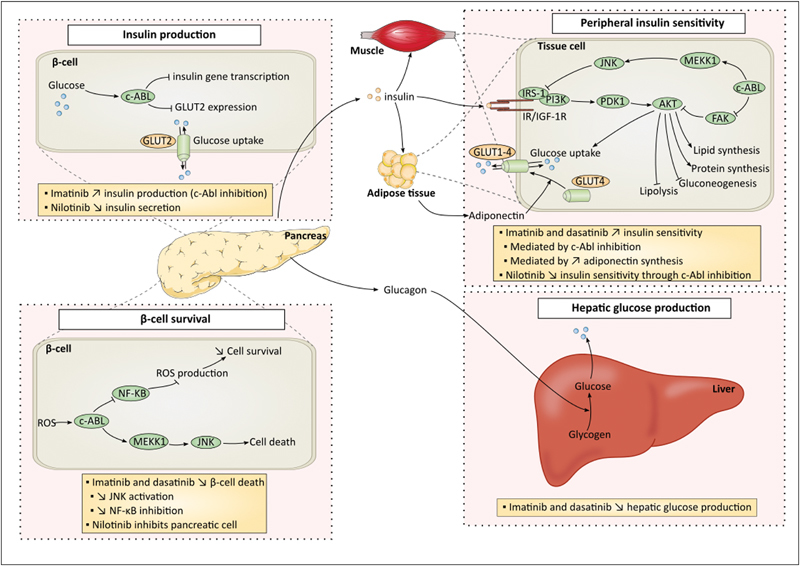
Effects of BCR-ABL TKIs on glucose metabolism. Imatinib and dasatinib possess hypoglycemic effects, whereas nilotinib increases blood glucose level and diabetes development. The figure describes glucose metabolism and boxes contain emitted hypotheses for effects of imatinib, dasatinib, and nilotinib on glucose metabolism. Four major hypotheses have been emitted including impact on insulin production by β-cells, β-cell survival, peripheral insulin sensitivity, and hepatic glucose production. ABL, Abelson; FAK, focal adhesion kinase; GLUT, glucose transporter; IRS-1, insulin receptor substrate 1; JNK, c-Jun N-terminal kinases; MEKK1, MAPK/ERK kinase kinase 1; NF-κB, nuclear factor-kappa B; PDK1, pyruvate dehydrogenase kinase 1; PI3K, phosphoinositide 3-kinase; ROS, reactive oxygen species.

BCR-ABL TKIs have contradictory effect on glucose metabolism. Imatinib and dasatinib improve glucose metabolism and type 2 diabetes management in CML patients (i.e., decrease of antidiabetic drug dosage and reversal of type 2 diabetes). 14 45 46 47 48 49 This clinical profile is in accordance with in vivo studies in which imatinib is effective to prevent the development of type 1 diabetes in prediabetic mice, without impacting the adaptive immune system. 50 Therefore, imatinib is currently tested in clinical trials for patients suffering from type 1 diabetes mellitus (NCT01781975). The mechanism(s) by which dasatinib and imatinib improve glucose metabolism remains unknown. Global hypotheses suggest that imatinib increases peripheral insulin sensitivity, promotes β-cell survival, or decreases hepatic glucose production ( Fig. 2 ). 51 52 53 54 This latter hypothesis (i.e., decreased hepatic glucose production by imatinib) is not currently the preferred theory, whereas it was demonstrated that imatinib weakly affects hepatic glucose production. 51 Several targets might be involved in this metabolic effect. PDGFR has already been linked with type 1 diabetes reversal. 50 Hägerkvist et al hypothesized that c-Abl inhibition by imatinib promotes β-cell survival through activation of NF-κB signaling and inhibition of proapoptotic pathways ( Fig. 2 ). 53 54 Inhibition of c-Abl in β-cells might also increase insulin production and contribute to the glucose regulation by imatinib. 55 It was also speculated that imatinib decreases insulin resistance in peripheral tissues due to c-Abl-dependent JNK inactivation. d 51 Similar hypotheses might be translated to dasatinib because of the similar off-target inhibitory profile (i.e., dasatinib also inhibits c-Abl and PDGFR). It was hypothesized that imatinib and dasatinib impact glucose metabolism through reduced adipose mass. 51 56 However, clinical data do not demonstrate weight loss in CML patients and do not favor this hypothesis. In both imatinib- and dasatinib-treated patients, increased circulating adiponectin e level correlates with decreased insulin resistance. 57 58 This correlation might be explained by the translocation of the glucose transporter GLUT4 f from the cytoplasm to the cell membrane following adiponectin signaling. 59 Additionally, adiponectin has been related to decreased hepatic glucose production which could be an additional mechanism by which imatinib and dasatinib improve glucose metabolism. 60 It was speculated that the raise of adiponectin level with imatinib and dasatinib is the consequence of increased adipogenesis subsequent to PDGFR inhibition. 61

Oppositely to imatinib and dasatinib, case reports and clinical trials indicate that nilotinib increases blood glucose level and promotes diabetes mellitus. 62 63 64 65 Indeed, 20% of nilotinib-treated patients developed diabetes after 3 years of treatment, 65 whereas 29% of patients suffer from increase of fasting glucose after 1 year of therapy. 64 However, no variations of glycated hemoglobin were reported. 64 65 Clinical data indicate no direct effect of nilotinib on β-cells, but suggest fasting insulin increase, fasting C-peptide decrease, and an increase of HOMA-IR values (i.e., a model to assess insulin resistance). 64 66 67 Therefore, the preferred hypothesis to explain the development of hyperglycemia is the manifestation of insulin resistance. Weakened insulin secretion occurred sometimes, but it is likely that this impairment is the consequence of β-cell exhaustion. 68 However, in vitro experiments demonstrated inhibitory effect of nilotinib on pancreatic cell growth. 69 Breccia et al proposed an additional hypothesis linking development of hyperglycemia and body mass index. They suggested that the development of hyperglycemia might be the consequence of increase fat level tissue resulting in decrease peripheral insulin sensitivity. 70 However, dietetic measures to restrict glucose exogenous uptake in patients who developed hyperglycemia were not successful, 63 and nilotinib does not induce changes in patient body weight. 71 Little is known regarding the mechanism by which nilotinib induces insulin resistance. Racil et al suggested that peripheral insulin resistance is mediated by c-Abl inhibition which is involved in insulin receptor signaling ( Fig. 2 ). 67 This hypothesis is contrary to the hypothesis described with dasatinib and imatinib in which c-Abl enhances insulin sensitivity through c-Abl inhibition. These two hypotheses describe different pathways involving c-Abl but with opposite outcomes. To date, no hypothesis is preferred and additional studies are required to understand the opposite effect on glucose metabolism between TKIs, whereas both have been attributed to c-Abl inhibition. Interestingly, Frasca et al described opposite role of c-Abl in insulin signaling depending on the receptor involved, the signaling pathway, and the cell context. 72 Similar investigations should be performed in the context of c-Abl inhibition by BCR-ABL TKIs. For bosutinib and ponatinib, little is known regarding their impact on glucose metabolism, but no drastic changes in glucose profile has been reported during clinical trials.

### Lipid Metabolism

**Fig. 3:**
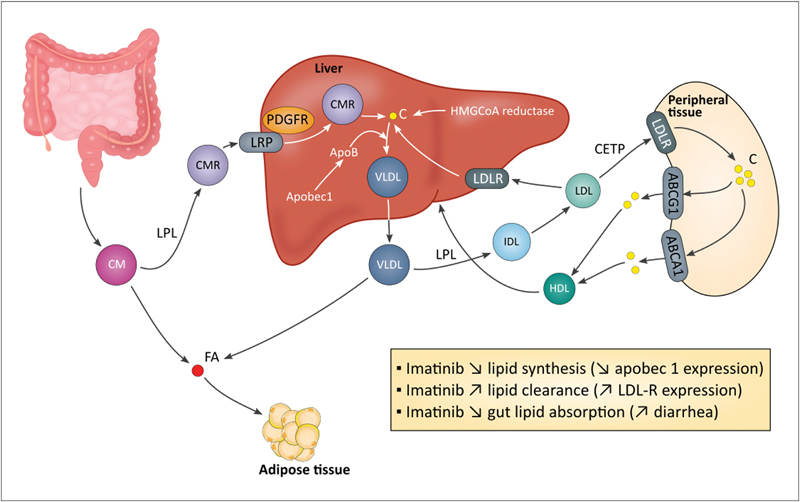
Effects of BCR-ABL TKIs on lipid metabolism. Several hypotheses have been emitted to explain the imatinib-induced hypolipidemic effect. Imatinib regulates expression of genes involved in lipid metabolism: Apobec1 that regulates ApoB expression through the introduction of a stop codon into ApoB mRNA (ApoB is essential for VLDL production), and LDLR that is implicated in lipid clearance. Imatinib-induced PDGFR inhibition influences LPL synthesis and dysregulates LRP. Dasatinib and nilotinib increase cholesterol plasma level through an unknown mechanism. Global hypotheses can be emitted and include increased hepatic lipid synthesis (possibly related to hyperinsulinemia) and decreased lipid clearance through LDLR functional defect or decreased LPL synthesis. ABC, ATP-binding cassette; C, cholesterol; CETP, cholesteryl ester transfer protein; CM, chylomicron; FA, fatty acid; HMGCoA reductase, hydroxymethylglutaryl-CoA reductase; IDL, intermediate-density lipoprotein; LDL, low-density lipoprotein; LDLR, low-density lipoprotein receptor; LPL, lipoprotein lipase; LRP, lipoprotein receptor-related protein; PDGFR, platelet-derived growth factor receptor; VLDL, very low-density lipoprotein.

Similarly with glucose metabolism, effects on lipid metabolism are conflicting between TKIs. Oppositely to in vivo study which demonstrated no impact of imatinib on total cholesterol and triglycerides levels in diabetic mice, 29 imatinib is associated in CML patients with a rapid and progressive decrease of cholesterol and triglycerides levels. 66 73 74 75 First hypothesis relates the inhibition of PDGFR by imatinib ( Fig. 3 ). PDGFR is involved in the synthesis of the lipoprotein lipase (LPL) and in the regulation of the lipoprotein receptor-related protein (LRP). 73 74 However, all BCR-ABL TKIs possess inhibitory activity against PDGFR but do not share this positive impact on lipid profile. Recently, Ellis et al described that imatinib impairs gene expression of proteins involved in plasma lipid regulation. Indeed, in in vitro model of CML cells, imatinib affects gene expression of four genes implicated in lipid synthesis (HMG-CoA reductase g gene and apobec1 h ), lipid clearance (LDLR gene i ) and in exchange of lipids from very low-density lipoprotein (VLDL) or low-density lipoprotein (LDL) to high-density lipoprotein (HDL) (CETP j gene). However, these studies were performed in a model of CML cells and need to be confirmed in more relevant models (e.g., primary cell lines, hepatocytes). 76 Franceschino et al suggested that imatinib decreases diarrhea-related lipid absorption due to inhibition of c-kit in interstitial Cajal cells (i.e., c-kit signaling is critical for the survival and development of these cells). 73 However, this hypothesis is unlikely, few patients (3.3%) developed grade 3/4 diarrhea, and patients treated with interferon-α and cytarabine developed diarrhea at a same rate and do not present lipid level reduction in the phase 3 clinical trial (NCT00333840).

Oppositely, dasatinib and mostly nilotinib are associated with an increase of cholesterol level. 26 66 77 Nilotinib induces quick rise of total cholesterol, HDL, and LDL (i.e., within 3 months). Nilotinib-induced dyslipidemia are responsive to statin and lipid level normalized after nilotinib discontinuation. 78 To date, the mechanism by which dasatinib and nilotinib impact lipid metabolism is unknown. Future researches should determine how these treatments induce dyslipidemia. Global hypotheses could be formulated and include an increase of lipid synthesis that might be secondary to insulin resistance and hyperinsulinemia. This hypothesis is particularly relevant with nilotinib and it is also associated with hyperglycemia. Dasatinib and nilotinib might also decrease blood lipid clearance (e.g., disturbance of LDLR and LPL synthesis). The development of dyslipidemia might contribute to the occurrence of arterial occlusive events that occurred with nilotinib and dasatinib. However, the relationship between impaired lipid metabolism and cardiovascular occlusive events is unknown with BCR-ABL TKIs, and there is no indication that correct management of lipid metabolism can prevent arterial thrombosis (e.g., stenosis occurred in a nilotinib-treated patient despite the management of its hyperlipidemia through statin treatment). 79 On their side, bosutinib and ponatinib do not disturb lipid metabolism. 78 80

## Effects on Atherosclerosis

### Endothelial Dysfunction

Fig. S1 in the Supplementary Material details the role of endothelial cells (ECs) in atherosclerosis. Several in vitro and in vivo experiments assess the impact of imatinib on EC viability and functions ( Table 2 ). These studies demonstrate that imatinib does not affect EC viability nor induce apoptosis but increases EC proliferation. 39 81 82 83 84 Only one study reports a proapoptotic effect of imatinib on ECs, but their experiments were performed on a cell line (i.e., EA.hy926 cells), 85 a model less reliable than primary cultures (e.g., HUVEC, k HCAEC l ). In vitro studies also assessed the effect of imatinib on EC functions. In these studies, imatinib does not influence adhesion molecule expressions (i.e., ICAM-1 m and VCAM-1 n ), EC migration, reactive oxygen species (ROS) production, nor angiogenesis. 81 82 85 86 87 Letsiou et al suggested that imatinib decreases EC inflammation by decreasing the secretion of proinflammatory mediators. 86 The impact of imatinib on endothelial permeability is not clear. Indeed, in vitro studies demonstrate that imatinib increases endothelial permeability by decreasing the level of plasma membrane VE-cadherin, o 85 86 whereas in vivo experiments indicate decreased vascular leak following imatinib treatment in a murine model of acute lung injury. 88 Additionally, imatinib has been tested in patients suffering from acute lung injury, a disease characterized by vascular leakage, and demonstrate promising clinical efficacy. Therefore, imatinib might positively affect atherogenesis by decreasing endothelial inflammation and reducing vascular leakage.

**Table 2:** In vivo and in vitro investigations of the effects of BCR-ABL TKIs on endothelial cell viability and major functions

Endpoints	Methods	Models	TKIs	Findings	Ref.
EC proliferation/survival	Cell counting; trypan blue staining	EA.hy 926 cell; HCAEC	Imatinib	= EC viability <10µM	84 85
	Caspase assay; Annexin V staining; Hoechst staining; TUNEL assay	HMEC-1; HUVEC; Human pulmonary EC; Mouse EC	Imatinib	= EC apoptosis	81 82 87
	TUNEL assay; Annexin V staining	EA.hy 926 cell	Imatinib	EC apoptosis	85
	MTT cell proliferation assay; ^3^ H-thymidine incorporation; WST-1 assay; cell counting	HMEC-1; HUVEC; HCAEC	Imatinib	= EC proliferation	39 81 82 84
	Resazurin proliferation assay; PCNA expression	HUVEC; BAEC	Imatinib	EC proliferation (≥1.2 µM)	83
	Caspase assay; Hoechst staining; Annexin V staining; TUNEL assay	Human pulmonary EC	Dasatinib	EC apoptosis	87
	^3^ H-thymidine incorporation; WST-1 assay; MTT assay	HUVEC; HCAEC; HMEC-1; HCtAEC	Nilotinib	EC proliferation	39 82 89
	Annexin V staining	HUVEC	Nilotinib	= EC apoptosis	82
	Caspase assay; Annexin V staining	HCAEC; HUVEC	Ponatinib	EC apoptosis	82 90
	^3^ H-thymidine incorporation; WST-1 assay	HUVEC; HMEC-1; EPC	Ponatinib	EC proliferation	82 90
Oxidative stress	Fluorescent ROS detection; Immunofluorescence (8-oxo-dG)	Human Pulmonary EC; Rat lung	Imatinib	= endothelial ROS	87
	Fluorescent ROS detection; Immunofluorescence (8-oxo-dG)	Human Pulmonary EC; Rat lung	Dasatinib	endothelial ROS	87
EC migration	Wound scratch assay; Microchemotaxis assay; Transwell migration assay	HMEC-1; HUVEC; EA.hy 926 cell; HCAEC	Imatinib	= EC migration	81 82 84 85
	Wound scratch assay	HUVEC; HCAEC; HMEC-1	Nilotinib	EC migration	39
	Transwell migration assay	HUVEC	Nilotinib	= EC migration	82
	Transwell migration assay	HUVEC	Ponatinib	EC migration	82
Angiogenesis	Tube-formation assay	HMEC-1; HUVEC	Imatinib	= angiogenesis	81 82
	Tube-formation assay	HUVEC; HCAEC; HMEC-1	Nilotinib	angiogenesis	39
	Tube-formation assay	HUVEC	Nilotinib	= angiogenesis	82
	Tube-formation assay	HUVEC	Ponatinib	angiogenesis	82
Permeability	Permeability to albumin	EA.hy 926 cell	Imatinib	endothelial permeability (10 µM)	85
	Immunofluorescence (VE-cadherin)	EA.hy 926 cell; HPAEC	Imatinib	membrane VE-cadherin (10 µM)	85 86
	BAL protein levels	Mice (2-hit model of ALI)	Imatinib	BAL protein levels	86 88
	Permeability to FITC-Dextran; permeability to HRP	HMEC-1; HUVEC; Human lung microvascular EC	Imatinib	= endothelial permeability	94 147
	Immunostaining	HUVEC	Imatinib	intercellular gaps	147
	Evans blue/albumin extravasation	Mice	Imatinib	Evans blue extravasation	147
	Pulmonary microvascular permeability assay; permeability assay (FITC-Dextran)	Mice; HMEC-1; HPAEC	Dasatinib	endothelial permeability	94
	Permeability assay (FITC-Dextran)	HRMEC	Dasatinib	VEGF-induced permeability	148
CAM expression	Confocal microscopy; ELISA; qRT-PCR; flow cytometry	HMEC-1; Pulmonary EC (rat lung); EA.hy926	Imatinib	= ICAM-1, VCAM-1 and E-selectin expression = soluble ICAM-1, VCAM-1 and E-selectin	81 87 149
	Immunoblotting (VCAM-1)	Human lung EC	Imatinib	VCAM-1 expression	86
	Confocal microscopy	Pulmonary EC (rat lung)	Dasatinib	ICAM-1, VCAM-1 and E-selectin expression	87
	ELISA	Rat	Dasatinib	soluble ICAM-1, VCAM-1 and E-selectin	87
	qRT-PCR; flow cytometry	EA.hy926	Dasatinib	= ICAM-1, VCAM-1 and E-selectin expression	149
	Unknown	HUVEC	Nilotinib	ICAM-1, VCAM-1 and E-selectin expression (≥1 µM)	39
	qRT-PCR; flow cytometry	EA.hy926	Nilotinib	ICAM-1, VCAM-1 and E-selectin expression	149
Secretory	ELISA (IL-6; IL-8)	Stimulated HPAEC	Imatinib	IL-8 and IL-6 (LPS induced)	86
	qRT-PCR ; ELISA (IL-1β; IL-6; TNF-α)	EA.hy926 cell ; HUVEC	Imatinib	= IL-1β, IL-6 and TNF-α expression and production	149
	qRT-PCR ; ELISA (IL-1β; IL-6; TNF-α)	EA.hy926 cell ; HUVEC	Dasatinib	= IL-1β, IL-6 and TNF-α expression and production	149
	qRT-PCR ; ELISA (IL-1β; IL-6; TNF-α)	EA.hy926 cell ; HUVEC	Nilotinib	= IL-6 and TNF-α expression and production IL-1β expression and production	149
	ELISA (t-PA; PAI-1; ET-1; vWF; total NO)	HCtAEC	Nilotinib	t-PA PAI-1, ET-1, vWF and total NO	89
Adhesion	Unknown	HUVEC	Ponatinib	adhesion to plastic surface at 1 µM	90

Abbreviations: 8-oxo-dG, 8-hydroxy-2′-deoxyguanosine; ALI, acute lung injury; BAEC, bovine aortic endothelial cell; BAL, bronchoalveolar level ; EC, endothelial cell; ELISA, enzyme-linked immunosorbent assay; EPC, endothelial progenitor cell; ET-1, endothelin 1; FITC, fluorescein isothiocyanate; HCAEC, human coronary artery endothelial cell; HCtAEC, human carotid artery endothelial cell; HMEC-1, human microvascular endothelial cell; HPAEC, human pulmonary artery endothelial cell; HRMEC, human retinal microvascular endothelial cells; HUVEC, human umbilical vein endothelial cell; ICAM-1, intercellular adhesion molecule 1; IL, interleukin; LPS, lipopolysaccharide; NO, nitric oxide; PAI-1, plasminogen activator inhibitor-1; ROS, reactive oxygen species; t-PA, tissue plasminogen activator; TUNEL, terminal deoxynucleotidyl transferase dUTP nick end labeling; VCAM-1, vascular cell adhesion molecule 1; VE-cadherin, vascular endothelial cadherin; vWF, Von Willebrand factor.

Nilotinib and ponatinib reduce EC proliferation and might impaired endothelial regeneration. 39 82 89 90 Additionally, ponatinib induces EC apoptosis, although it is well recognized that high glucose concentration induces EC death, 91 suggesting that nilotinib might, by this intermediary, affect EC viability. Moreover, clinical data indicate that dasatinib induces pulmonary arterial hypertension, whereas imatinib is possibly beneficial in this disease. 92 93 This pathology is initiated by dysfunction or injury of pulmonary ECs. 87 Therefore, in vivo and in vitro studies investigated effect of imatinib and dasatinib on pulmonary ECs and demonstrate that dasatinib induces apoptosis on pulmonary ECs mediated by increased mitochondrial ROS production. 87 Future researches should assess if this effect is also found in arterial ECs and ROS production should also be tested with other new-generation BCR-ABL TKIs.

In addition to their effect on EC viability, nilotinib and ponatinib also influence EC functions, inhibit their migration, and decrease angiogenesis. 39 82 It was suggested that the antiangiogenic effect of ponatinib is the consequence of VEGFR p inhibition, but this hypothesis cannot explain the antiangiogenic effect of nilotinib (i.e., nilotinib does not inhibit VEGFR). 82 Nilotinib also increases adhesion molecule expressions (i.e., ICAM-1, VCAM-1, and E-selectin) in vitro, 39 suggesting that nilotinib might increase leukocyte recruitment. However, further experiments are needed to validate this hypothesis (e.g., assessment of endothelium permeability and transendothelial migration). Dasatinib also induces endothelium leakage in vitro, and the RhoA-ROCK q pathway is involved in this phenomenon. 94 It was demonstrated that RhoA activation induces the phosphorylation of myosin light chain that increases the actomyosin contractibility and disrupt endothelial barrier. 94 Therefore, increased endothelium permeability is a potential mechanism by which dasatinib and nilotinib promote atherosclerosis development and arterial thrombosis. Likewise, it is plausible that ponatinib affects endothelium integrity because of its inhibitory activity against VEGFR, which is recognized as a permeability-inducing agent. Additional hypotheses suggest that inhibition of Abl kinase (i.e., Arg r and c-Abl) and PDGFR might also be implicated in vascular leakage. 85 Finally, Guignabert et al demonstrated that both in rats and in CML patients taking dasatinib, there is an increase of soluble adhesion molecules, which are well-known markers of endothelial dysfunction. 87

### Inflammation

Fig. S2 in the Supplementary Material describes the role of immune cells and inflammation process during atherosclerosis. Table 3 summarizes in vitro studies that investigate impacts of BCR-ABL TKIs on survival, proliferation, and major functions of monocytes, macrophages, and T-lymphocytes. Globally, in vitro studies demonstrate that imatinib inhibits the development and maturation of monocytes and alters monocyte functions. 95 96 Imatinib decreases production of proinflammatory cytokines (i.e., TNF-α s and IL-6 t ) and diminishes the potential of monocytes to phagocytose. 97 98 These impacts on monocyte functions are possibly related to c-fms u inhibition. 99 Imatinib also inhibits macrophage functions in vitro. Imatinib decreases lipid uptake without impacting the lipid efflux and decreases activity and secretion of two matrix metalloproteinases (MMPs; i.e., MMP-2 and MMP-9 v ) on a posttranscriptional level. 100 Additionally, imatinib inhibits T-lymphocyte activation and proliferation and decreases proinflammatory cytokines secretion (i.e., IFN-γ w ). 101 The inhibition of monocyte, macrophage, and T-cell functions by imatinib might prevent the development of atherosclerosis or reduce the risk of atherosclerotic plaque rupture.

**Table 3:** In vitro studies on effects of BCR-ABL TKIs on proliferation, survival, and major functions of monocytes, macrophages, and T-lymphocytes

Endpoints	Methods	Models	TKIs	Findings	Ref.
**Monocytes/Macrophages**					
Proliferation/survival	Propidium iodide staining	PBMC	Imatinib	= viability	150
	Cell counting	Ovarian tumor ascites samples	Imatinib	macrophage production	96
	Cell counting	Ovarian tumor ascites samples	Dasatinib	macrophage production	96
	WST-1 assay	Human macrophages	Ponatinib	= macrophage viability	82
Monocyte differentiation	Morphology assessment	Human monocyte	Imatinib	differentiation into macrophages	95
Secretion	ELISA; qPCR	Human monocyte and macrophage; PBMC	Imatinib	TNF-α, IL-6 and IL-8 production	97 150
	ELISA	PBMC; Human monocyte and macrophage	Imatinib	= IL-10 production	150
	ELISA; Bioplex system; nitrite assay	Raw 264.7; bone-marrow derived macrophage	Dasatinib	TNF-α, IL-6, IL-12p40 and NO production	103 151
	qPCR; Bioplex system	Primary macrophage (mice)	Dasatinib	IL-10 production	103
	Bioplex system	Bone-marrow derived macrophage	Bosutinib	IL-6, IL-12p40 and TNF-α production	103
	qPCR; Bioplex system	Primary macrophage (mice)	Bosutinib	IL-10 production	103
Phagocytosis	Antigen-uptake assay	Human monocyte	Imatinib	phagocytosis	97
Cholesterol uptake	Cholesterol uptake assay	THP-1; PBMC	Imatinib	LDL uptake	100
	Cholesterol uptake assay	THP-1	Bosutinib	LDL uptake	100
MMP production/activity	Zymography	THP-1	Imatinib	MMP-2 and MMP-9 secretion and activity	100
**T Lymphocytes**					
Proliferation/survival	^3^ H-TdR incorporation; CFSE staining; titrated thymidine	Naïve CD4 ^+^ T cell; Human T cell	Imatinib	T-cell proliferation	101 152 153
	Annexin V staining; Caspase assay	Human T cell	Imatinib	= T-cell apoptosis	101 152 153
	Annexin V staining	Human T cell	Imatinib	= T cell apoptosis	
	CFSE dye	Human T cell	Dasatinib	T-cell proliferation	107
	Annexin V staining	PBMC; Human T cell	Dasatinib	= T cell viability	105 107
	CFSE dye	CD8 ^+^ T cell; PBMC	Nilotinib	T cell proliferation	106 154
Secretion	ELISA	Human T cell; CD8 ^+^ and CD4 ^+^ T cell	Imatinib	IFN-γ production	101 107
	ELISA; proteome profile array	Human T cell; PBMC	Dasatinib	TNF-α, IFN-γ, IL-2, IL-6, IL-17 production	105 107
	Proteome profile array	PBMC	Dasatinib	chemotactic factors secretion (SDF-1, MIP-1α, MIP-1β, MCP-1, CXCL-1)	105
	ELISPOT assay	CD8 ^+^ T cell	Nilotinib	IFN-γ production	154
Activation	Immunofluorescence	Human T cell	Imatinib	T cell activation	101
	Flow cytometry (CD25, CD69)	Human T cell	Imatinib	= T cell activation	153
	Flow cytometry (CD25, CD69)	Human T cell; PBMC	Dasatinib	T cell activation	105 107
	Flow cytometry (CD25, CD69)	Human T cell	Nilotinib	T cell activation	154

Abbreviations: CFSE, carboxyfluorescein succinimidyl ester; CXCL1, (C-X-C motif) ligand 1; ELISA, enzyme-linked immunosorbent assay; ELISPOT, enzyme-linked immunospot; IFN, interferon; IL, interleukin; MCP, monocyte chemoattractant protein-1; MIP-1, macrophage inflammatory protein 1; NO, nitric oxide; PBMC, peripheral blood mononuclear cell; qPCR, quantitative polymerase chain reaction; SDF-1, stromal cell-derived factor 1; TNF, tumor necrosis factor.

Effects of new-generation TKIs on inflammatory cells were less studied, but first experiments indicate similarities with imatinib about its impact on monocytes and macrophages. Both dasatinib and nilotinib have similar inhibitory profile on macrophage-colony formation that has been linked to CSFR inhibition. 96 102 Dasatinib also possesses anti-inflammatory functions by attenuating proinflammatory cytokines production (i.e., TNF-α, IL-6, and IL-12 x ) by macrophages and increasing production of anti-inflammatory mediator (i.e., IL-10 y ). 103 These effects are thought to be mediated by SIK z inhibition, a subfamily of three serine/threonine kinases that regulate macrophage polarization. 103 104 Finally, dasatinib is associated with decreased T-cell functions and particularly it decreases the production of proinflammatory cytokines (e.g., TNF-α, IFN-γ) and chemotactic mediators. 105 Nilotinib and bosutinib also possess anti-inflammatory activity and decrease cytokine production and T-cell activation. 103 106 Inhibition of Lck, ai a tyrosine kinase implicated in T-cell receptor signaling, is implicated in the impairment of T-cell functions by dasatinib and nilotinib. 107 108 It has been hypothesized that nilotinib decreases mast cell activity through c-kit inhibition, 62 109 which might result in a decrease of the vascular repair system. 39 62 Clinical profile of nilotinib in patients with CML consolidates this hypothesis and demonstrates a decreased of mast cell level. 39 However, similar decreased of mast cell is also reported with imatinib without high rate of arterial thrombosis. 110

Globally, BCR-ABL TKIs possess reassuring profile on inflammatory cells. However, impact of new-generation TKIs on several functions of macrophages have not been assessed (e.g., MMP secretion and activity, lipid uptake, and foam cell formation), whereas effect of ponatinib on inflammatory cells is unknown. The assessment of lipid uptake and foam cell formation is particularly relevant with new-generation TKIs because there are numerous interactions between TKIs and ABC transporters. aii 111 112

### Fibrous Cap Thickness

Fig. S3 in the Supplementary Material describes the mechanism by which atherosclerotic plaque ruptures and induces arterial thrombosis. Table 4 summarizes in vitro and in vivo experiments performed on VSMCs and fibroblasts. Imatinib decreases VSMC proliferation and growth but results are conflicting about its impact on apoptosis. Some studies demonstrate no impact on SMC apoptosis, whereas others indicate increased SMC death. 83 113 114 115 116 Imatinib also affects VSMC functions and decreases their migration and LDL binding, inducing decreased LDL retention by the sub-endothelium. 113 117 Imatinib also exerts negative effect on the synthesis of major ECM components (type I collagen and fibronectin A) by fibroblasts, correlating to decreased ECM accumulation in vivo. 118 The impact of imatinib on SMCs is thought to be mediated by PDGFR inhibition, 114 which is involved in several VSMC functions including VSMC survival and plasticity. 113 Subsequent to the hypothesis that imatinib inhibits PDGFR signaling, prevents abundant SMC and fibroblast proliferation, and inhibits abundant ECM accumulation, imatinib has been tested for the management of several fibrotic diseases (e.g., dermal and liver pulmonary fibrosis, systemic sclerosis). 30 118 119 Imatinib successfully acts on pulmonary fibrosis and pulmonary arterial hypertension (i.e., a disease involving vascular remodeling mediated by pulmonary SMC proliferation), 93 114 and has beneficial activity in sclerotic chronic graft-versus-host disease. 120 Finally, imatinib was tested in vivo for the prevention of cardiovascular diseases and demonstrates efficacy for the treatment of myocardial fibrosis by reducing ECM component synthesis (i.e., procollagen I and III). 30 In a rat model, imatinib successfully inhibits stenosis after balloon injury and presents interest in intimal hyperplasia and stenosis after bypass grafts. 115 116 121 122 123 Imatinib also successfully prevents arterial thrombosis following microvascular surgery in rabbits. 124 Imatinib was also encompassed in a stent but do not demonstrate efficacy in restenosis prevention. 84

**Table 4:** In vitro and in vivo studies on effects of BCR-ABL TKIs on proliferation, survival, and major functions of smooth muscle cells and fibroblasts

Endpoints	Methods	Models	TKIs	Findings	Ref.
Proliferation/survival	Resazurin assay; immunofluorescence; ^3^ H-thymidine incorporation; BrdU incorporation; MTT assay	HVSMC; BAoSMC; PASMC; ASMC; VSMC; HAoSMC; HCASMC; Rabbit	Imatinib	SMC proliferation	83 84 114 115 116 123 155
	Caspase assay; PARP (Western blot); JC-1 dye; Annexin V staining	BAoSMC; Dermal fibroblast; PASMC	Imatinib	= SMC/fibroblast apoptosis	83 118 155
	TUNEL; caspase assay	PASMC; HAoSMC; Rabbit	Imatinib	SMC apoptosis (PDGF-stimulated)	114 116 123
	Trypan blue exclusion	HCASMC; A10 cell line	Imatinib	= SMC viability	84
	Cell counting; Propidium iodide staining	A10 cell line, HAoSMC	Dasatinib	SMC proliferation	113 125
Migration	Transwell cell migration assay	HAoSMC; PASMC; HCASMC; A10 cell	Imatinib	SMC migration	84 116 155
	Transwell cell migration assay	HAoSMC; A10 cell	Dasatinib	SMC migration	113 125
Secretion/synthesis	Radiolabel incorporation	Human VSMC	Imatinib	proteoglycan synthesis	117
	RT-PCR; Western blot; Sircol collagen assay	Dermal fibroblast	Imatinib	COL1A1, COL1A2, fibronectin 1 synthesis collagen synthesis	118
	RT-PCR	Dermal fibroblast	Imatinib	= MMP-1, MMP-2, TIMP-1, TIMP-2, TIMP-3 and TIMP-4	118
	qRT-PCR	Human fibroblast	Nilotinib	Decreases COL1A1 and COL1A2 synthesis	127
Fibrosis	Sirius red staining	Rat	Imatinib	myocardial fibrosis, liver fibrosis	30 119
	Intima/media ratio	Rat (Balloon injury model)	Imatinib	stenosis	121 122
	Intima/media ratio	Rabbit	Imatinib	intimal thickness	124
	Hydroxyproline, collagen content	Rat liver	Imatinib	hydroxyproline and collagen content	128
	Hydroxyproline, collagen content	Rat liver	Nilotinib	hydroxyproline and collagen content	128
	Sirius red staining	Rat liver	Nilotinib	liver fibrosis	128

Abbreviations: ASMC, arterial smooth muscle cell; BAoSMC, bovine aortic smooth muscle cell; BrdU, bromodeoxyuridine; COL, collagen; HaOSMC, human aortic smooth muscle cell; HCASMC, human coronary artery smooth muscle cell; HVSMC, human vascular smooth muscle cell; MMP, matrix metalloproteinase; PARP, poly(ADP-ribose) polymerase; PASMC, pulmonary smooth muscle cell; PDGF, platelet-derived growth factor; qRT-PCR, quantitative reverse transcription polymerase chain reaction; SMC, smooth muscle cell; TIMP, tissue inhibitor of metalloproteinase; TUNEL, terminal deoxynucleotidyl transferase dUTP nick end labeling; VSMC, vascular smooth muscle cell.

Impact of new-generation TKIs on fibrosis was less studied but demonstrate similar inhibitory effect on VSMCs and fibroblasts. Indeed, dasatinib inhibits PDGFR more potently than imatinib, 113 and the hypothesis that dasatinib prevents restenosis similarly with imatinib was emitted. Therefore, a patent has been filed claiming the use of dasatinib for the prevention of stenosis and restenosis. 125 Compared with imatinib, dasatinib has additional off-targets and is able to inhibit Src, aiii a kinase involved in dermal fibrosis in addition to PDGFR. 126 Therefore, dasatinib was tested in patients with scleroderma-like chronic graft-versus-host disease, a disease resulting from inflammation and progressive fibrosis of the dermis and subcutaneous tissues, and first results are encouraging. 126 Nilotinib also appears to be clinically efficient in scleroderma-like graft-versus-host disease by reducing collagen expression. 127 Finally, nilotinib was tested in vivo for the treatment of liver fibrosis and demonstrates decreased fibrotic markers and inflammatory cytokines (IL-1α, IL-1β, IFN-γ, IL-6). 128 However, only low-dose nilotinib was found to be efficient against fibrosis and normalized collagen content. 128 This lack of antifibrotic effect at higher doses might be explained by inhibition of additional off-targets by nilotinib that affect the benefit of low-dose nilotinib against fibrosis. Arterial thrombosis occurring with dasatinib and nilotinib are probably not the consequence of VSMC impairment, but investigations should be performed on VSMCs rather than on fibroblasts. Additional investigations are warranted to complete impact of BCR-ABL TKIs on VSMC functions (e.g., VSMC apoptosis, proliferation, and migration) and confirm their safety toward VSMCs.

## Off-targets

**Fig. 4:**
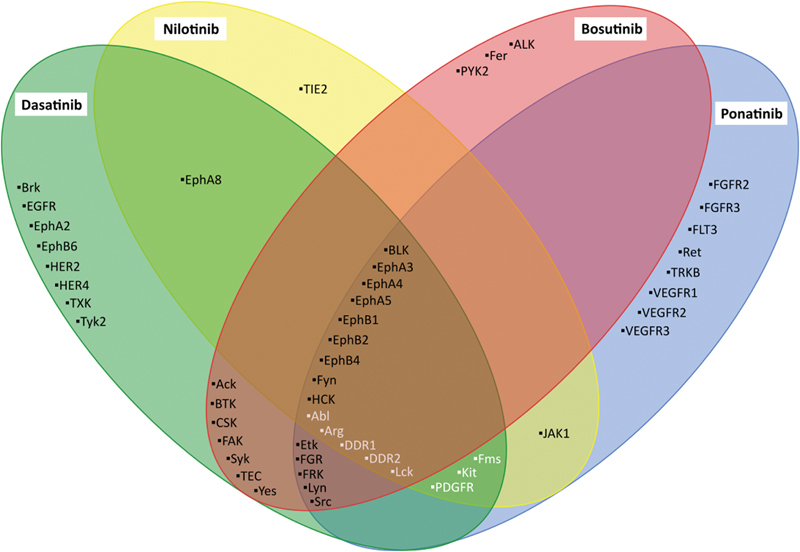
Specificity of imatinib, dasatinib, nilotinib, and ponatinib toward tyrosine kinases. Green, yellow, red, and blue circles contain tyrosine kinase inhibited by dasatinib, nilotinib, bosutinib, and ponatinib, respectively. Tyrosine kinases in white represent imatinib off-targets. This figure summarizes results from 13 experiments. 39 43 130 132 133 134 135 136 137 156 157 158 159 In case of conflictual results between studies, a conservative approach has been applied. Additional information is provided in the Supplementary Material .

BCR-ABL TKIs bind the highly conserved ATP binding site and are therefore not very specific to BCR-ABL and possess multiple cellular targets (kinases and nonkinase proteins). 129 130 This allowed the possibility to exploit them in other indications (e.g., PDGFR inhibition by imatinib is used in BCR-ABL-negative chronic myeloid disorders), 131 but this may also induce toxicities and side effects. 129 The development of arterial thrombotic events with new-generation BCR-ABL TKIs is likely to be related to inhibition of off-targets, as described throughout this review. Fig. 4 describes inhibitory profiles of imatinib, dasatinib, nilotinib, bosutinib, and ponatinib. Globally, imatinib is the most selective BCR-ABL TKIs, whereas dasatinib and ponatinib inhibit numerous off-targets.

However, inhibitory profiles are difficult to determine and several researches published discrepancies. For conflicting results, a conservative approach has been applied in Fig. 4 , but supplementary information ( Table S2 ) describes the tyrosine kinase selectivity profile of the five BCR-ABL TKIs and indicates divergences between studies. 43 130 132 133 134 These discrepancies can be explained by the difference in drug concentration and methodologies. To date, several methods have been used to determine inhibitory profile of BCR-ABL TKIs including in vitro kinase assay, 133 134 135 kinase expression in bacteriophages, 136 and affinity purification methods combined with mass spectrophotometry. 130 132 However, all these methods suffer from caveats, including the incompatibility to perform live-cell studies. A cell-permeable kinase probe was developed to figure out this problem, but this assay is still limited by the number of off-target tested (i.e., it requires to predefine tested off-targets) and therefore, the missing of targets is possible. 137 For this reason, the inhibitory activity of each TKI has not been tested toward all tyrosine kinase and Fig. 4 includes only off-targets for which at least one of the five BCR-ABL TKI has been tested. Thus, inhibitory profiles need to be carefully considered and it has to keep in mind that BCR-ABL TKI metabolites may possess activity against supplemental off-targets.

As described over this review, PDGF signaling has countless effects on several cells and tissues and is involved in several proatherogenic mechanisms (e.g., adipogenesis, vascular leakage, VSMC viability, and functions) and vascular homeostasis, which led to the suggestion of its implication in the potential beneficial cardiovascular effect of imatinib. 116 123 138 However, dasatinib, nilotinib, and ponatinib also inhibit PDGFR but increase the risk of arterial occlusive events. This difference of clinical outcome might be explained by the concentration of BCR-ABL TKIs necessary to obtain a same degree of PDGFR inhibition. 43 Indeed, Rivera et al reported that when adjusted to the maximum serum concentration, imatinib inhibits more profoundly PDGFR than dasatinib, nilotinib, and ponatinib. 43 Therefore, at effective concentration, it is probable that the degree of PDGFR inhibition is too low with dasatinib, nilotinib, and ponatinib to obtain the beneficial effect of PDGFR inhibition on atherosclerosis. Another possible hypothesis concerns the less conclusive specificity of new-generation TKIs which leads to inhibition of additional off-targets that might counterbalance the positive effect of PDGFR inhibition.

Other tyrosine kinases have been incriminated in the occurrence of arterial thrombosis with new-generation TKIs. DDR-1 aiv possesses functions in vascular homeostasis, atherogenesis, and is expressed in pancreatic islet cells. However, and similarly with PDGFR, it is inhibited by all BCR-ABL TKIs. 26 62 Other hypotheses include impairment of VEGF signaling by ponatinib 43 90 or the inhibition of several ephrin receptors by new-generation TKIs but not by imatinib which might inhibit monocyte recruitment. 139 Finally, it has been suggested that the inhibition of c-Abl itself is implicated in the increase of the cardiovascular risk. Indeed, imatinib possesses lower inhibitory effect on c-Abl than new-generation TKIs, which might further explain the difference in cardiovascular safety. 43 Additionally, c-Abl modulates Tie-2, av a tyrosine kinase that possesses important effect on endothelial cell function, angiogenesis, and inflammation. 140 141

## Perspectives and Conclusions

This review summarizes the data underlying the potential preventive effect of imatinib on the occurrence of arterial thrombosis. Globally, in vitro and in vivo experiments demonstrate that imatinib possesses antiplatelet activity, hypolipidemic and hypoglycemic effects, and inhibits inflammation and atherosclerosis development in several cell types (i.e., decreases of inflammatory cell and VSMC functions and increased vascular permeability). These benefits were largely attributed to PDGFR inhibition. It is currently unknown why new-generation TKIs that also inhibit PDGFR present opposite cardiovascular safety profile and this point needs to be elucidated.

New-generation BCR-ABL TKIs increase the risk of arterial thromboembolism with different clinical features (e.g., time-to-event and absolute rate) and are associated with different safety profiles, suggesting different pathways to explain the pathophysiology. The safety profile of nilotinib is mostly characterized by impaired glucose and lipid metabolism. However, both the molecular mechanism of these alterations and their impact on the occurrence of arterial thrombosis are unknown. Both dasatinib and ponatinib exhibit antiplatelet effect, whereas it was recently suggested that nilotinib potentially induces prothrombotic phenotype of platelets. Based on the clinical characteristics and case reports, atherosclerosis appears the most plausible mechanisms by which new-generation TKIs induce arterial thrombosis. However, in vitro and in vivo studies of viability and functions of SMCs and inflammatory cells demonstrate reassuring impact of dasatinib and nilotinib, even if additional studies are required to complete this evaluation. However, first experiments indicate that dasatinib, nilotinib, and ponatinib influence EC survival and/or endothelium integrity, suggesting a reasonable hypothesis by which new-generation TKIs induce atherosclerosis development and, subsequently, arterial thrombosis. Additional studies on the shedding of functional extracellular vesicles by endothelial cells might be interesting regarding their important role in coronary artery diseases. 142 Finally, the impact of new-generation TKIs on human blood coagulation and fibrinolysis has never been studied and should be addressed.

To conclude, new-generation TKIs increase the risk of arterial thrombosis in patients with CML, whereas imatinib, the first-generation TKI, might prevent the development of cardiovascular events. To date, the cellular events and signaling pathways by which these events occurred are unknown and researches are extremely limited focusing mainly on imatinib and nilotinib. Researches need to be extended to all new-generation BCR-ABL TKIs (i.e., dasatinib, bosutinib, and ponatinib). The understanding of the mechanisms by which new-generation BCR-ABL TKIs induce or promote arterial occlusive events will improve the clinical uses of these therapies. To date, only general risk minimization measures have been proposed (e.g., management of dyslipidemia, diabetes, arterial hypertension following standard of care). 14 22 23 143 144 145 146 The understanding of the pathophysiology is required to implement the most appropriate risk minimization strategies for thrombotic events and to select patients to whom the prescription of these drugs should be avoided when applicable. Finally, the understanding of the pathophysiology will help in the design of new BCR-ABL inhibitors sparing the toxic targets.

## Review Criteria

Relevant articles published from the database inception to July 11, 2017, were identified from an electronic database (PubMed) using the keywords “vascular,” “thrombosis,” “atherosclerosis,” “arteriosclerosis,” “venous,” “arterial,” “hemostasis,” “metabolic,” “metabolism,” “glycemia,” “glycaemia,” “cholesterol,” “triglycerides,” and “platelet” combined with the five approved BCR-ABL TKIs. The search strategy is presented in supplementary files. Articles published in languages other than English were excluded from the analysis. Primary criteria were pathophysiological explanation of arterial thrombotic events. Abstracts and full-text articles were reviewed with a focus on atherogenesis, plaque rupture, platelet functions, and their link with the development of arterial thrombosis with BCR-ABL TKIs. The reference section of identified articles was also examined.

## Footnotes

a
*T315I: Substitution at position 315 in BCR-ABL from a threonine to an isoleucine. This substitution alters the structure of the ATP-binding pocket and eliminates a crucial hydrogen bond required for binding of first- and second-generation TKIs.*

b
*Ephrin family: Members of this family are involved in platelet spreading, adhesion to fibrinogen and platelet secretion.*

c
*PAR-1: protease-activated receptor 1. PAR receptors mediate cellular effects of thrombin in platelets and endothelial cells.*

d
*JNK: c-Jun N-terminal kinases. JNK is responsive to stress stimuli and mediates insulin resistance through inhibition of insulin receptor substrate.*

e
*Adiponectin is a protein regulating glucose metabolism. Adiponectin increases peripheral insulin sensitivity by improving glucose uptake.*

f
*GLUT4: Glucose transporter type 4. GLUT4 is an insulin-regulated glucose transporter expressed in peripheral tissues.*

g
*HMGCoA reductase: 3-hydroxy-3-methyl-glutaryl-coenzyme A reductase. HMGCoA reductase catalysis is the conversion of HMG-CoA to mevalonic acid, an essential step in cholesterol synthesis.*

h
*Apobec1: Apolipoprotein B mRNA editing enzyme catalytic subunit 1. Apobec1 introduces a stop codon into ApoB mRNA.*

i
*LDLR: Low-density lipoprotein receptor. This cell surface receptor mediates LDL endocytosis.*

j
*CETP: Cholesteryl ester transfer protein.*

k
*HUVEC: Human umbilical vein endothelial cells.*

l
*HCAEC: Human coronary artery endothelial cells.*

m
*ICAM-1: Intercellular adhesion molecule 1. ICAM-1 stabilizes leukocyte-endothelial cell adhesion and facilitates leukocyte transmigration.*

n
*VCAM-1: Vascular cell adhesion molecule 1. VCAM-1 mediates rolling-type and firm adhesion of leukocyte.*

o
*VE-cadherin: Vascular endothelial cadherin. VE-cadherin is a cell–cell adhesion molecules and implies in endothelial junctions.*

p
*VEGFR: Vascular endothelial growth factor. This protein plays major roles in vasculogenesis and angiogenesis.*

q
*RhoA-ROCK: Ras homolog gene family, member A—Rho-associated protein kinase. Rho-kinase regulates cytoskeletal reorganization, cell migration, cell proliferation, and survival.*

r
*Arg: Abelson-related gene (also known as ABL2). Arg possesses cytoskeletal-remodeling functions.*

s
*TNF-α: Tumor necrosis factor alpha. This cytokine is mainly involved in systemic inflammation and regulates immune cells.*

t
*IL-6: Interleukin 6. IL-6 is a proinflammatory cytokine secreted by T-cells and macrophages to stimulate immune response.*

u
*CSFR: Colony-stimulating factor receptor. CSFR drives growth and development of monocytes.*

v
*MMP-2 and MMP-9 are two proteases capable of degrading extracellular matrix components. These two MMPs are the main proteases involved in atherogenesis.*

w
*IFN-γ: Interferon gamma. IFN-γ is involved in innate and adaptive immunity and activates macrophages.*

x
*IL-12: Interleukin 12. IL-12 is involved in T-cell differentiation and functions.*

y
*IL-10: Interleukin 10. IL-10 exerts immunoregulation and regulates inflammation.*

z
*SIK: Salt-inducible kinase. SIKs regulate production of anti- and proinflammatory cytokines.*

ai
*Lck: Lymphocyte-specific protein tyrosine kinase. Lck is mostly involved in T-cell maturation.*

aii
*ABC transporter: ATP-binding cassette transporters. ABCG1 and ABCA1 are implicated in macrophage reverse cholesterol transport. Their deficiency leads to foam cell formation and atherosclerosis development.*

aiii
*Src is involved in angiogenesis and cell survival and proliferation.*

aiv
*DDR-1: Discoidin domain receptor tyrosine kinase 1. DDR1 is involved in the regulation of cell growth, differentiation, and metabolism.*

av
*Tie-2: Tunica interna endothelial cell kinase. Tie-2 regulates angiogenesis, endothelial cell survival, proliferation, migration, adhesion and cell spreading, cytoskeleton reorganization, and vascular quiescence. Tie-2 also possesses anti-inflammatory functions by preventing the leakage of proinflammatory mediators and leukocytes.*
