# Supplementary material for: BCR-ABL Tyrosine Kinase Inhibitors: Which Mechanism(s) May Explain the Risk of Thrombosis?
Source: TH Open. 2018 Feb 14;2(1):e68–88. doi: 10.1055/s-0038-1624566 (PMC6524858; doi:10.1055/s-0038-1624566)
Supplement: Supplementary file 1 — Supplementary Material [file 10-1055-s-0038-1624566-s170017.pdf]

Clinical Characteristics of Vascular Occlusive Events with BCR-ABL TKIs

►**Table S1** summarizes time and location characteristics of arterial occlusive events associated with BCR-ABL tyrosine kinase inhibitor (TKIs). A link exists between location of the obstructed artery and the clinical presentation. With dasatinib, nilotinib, and ponatinib, arterial occlusive events occurred preferentially in the cardiovascular circulation leading to myocardial infarction and angina pectoris.[1–7] However, numerous patients also developed ischemic events in both the peripheral and cerebral circulation. To our knowledge, the etiology of these events is unknown. Peripheral and cerebral occlusive events might be the result of embolism or might be the consequence of on-site atherosclerotic lesion rupture/erosion.[8] The differentiation between the two is difficult to establish because both of them shared similar clinical pictures. It has to be noted that arterial emboli can arise both from aortic atherosclerotic lesions and from atrial fibrillation. Nilotinib and ponatinib have been both associated with common occurrence of atrial fibrillation, whereas these events occurred rarely with imatinib.[9–11] Nevertheless, no causal relationship has been established and the incidence of drug-induced atrial fibrillation is too low to be responsible for the majority of arterial occlusive events with these two treatments. Remarkably, numerous patients taking nilotinib developed peripheral arterial occlusive disease (PAOD) with preferential tropism for lower limbs and small vessel, indicating a pathology close to those found in diabetic patients, and potentially linking nilotinib-induced diabetes to the development of peripheral occlusive events.[12,13] This indicate that PAOD occurring with nilotinib might be the result of atheroemboli that come from atherosclerotic lesions and are formed by small cholesterol crystal particles, rather than arterial emboli.[8]

However, even if the three new-generation TKIs are close regarding the location of arterial thrombosis, there are discrepancies regarding the delay between the initiation of therapy and the development of cardiovascular events (►**Table S1**). For ponatinib, arterial occlusive events developed quickly and are more frequent during the first and the second years of treatment (14.5 and 14.1% respectively) than after (10.5% during the third year and 7.2% after 3 years).[14] However, it appears unclear if this reduction over time is real or is an artifact due to ponatinib dose reduction (i.e., recommendations were provided by authorities to decrease ponatinib doses in clinical trials to minimize the risk of blood clot).[15] Nilotinib-associated cardiovascular events developed less quickly and the rate of these events is constant over time with gradual increase of cardiovascular events and no plateau obtain after 100 months of treatment.[7] It takes longer in young patients and in patients without prior risk factors to develop an arterial thrombotic events with nilotinib,[16] fostering the hypothesis of progressive development of stenosis or progression of preexisting atherosclerotic lesions. For imatinib, dasatinib, and bosutinib, few data are available. One report described short time-to-event with dasatinib,[17] whereas the rare arterial occlusive events in patients treated with imatinib occur very lately.[7]

►**Table S1** also reports risk factors linked to the risk of arterial thromboembolism with new-generation BCR-ABL TKIs. Cardiovascular risk factors are difficult to highlight due to the small size of the population in clinical trials and the selection bias (e.g., older patients are usually excluded from clinical trials). However, several analyses demonstrated that arterial occlusive events occurred preferentially in nilotinib-treated patients with preexisting atherosclerotic risk factor(s) (i.e., arterial hypertension, overweight, smoking, hypercholesterolemia, diabetes mellitus) rather than in patients with no comorbidities, but that neither antiplatelet drugs nor lipid-lowering treatments seem to prevent the occurrence of these events.[6,7,16,18] Similarly, atherothrombotic events in ponatinib-treated patients also occurred preferentially in patients with cardiovascular risk factors. In correlation with the occurrence of arterial thrombotic events mostly in high-risk patients, case reports raised the concern that PAOD occurred preferentially on pathological arteries. Indeed, several case reports described nilotinib-associated PAOD resistant to surgical and medical treatment (i.e., stent implantation, aspirin), suggesting a negative impact of nilotinib on preexisting atherosclerotic lesions, and possibly on vascular endothelium compliance.[19–22] Symptoms of PAOD usually improve when nilotinib is discontinued, without surgery.[23] An autopsy of a nilotinib-treated patient subsequently to myocardial infarction also revealed obliterating artery disease (i.e., obliterating coronary sclerosis and atherosclerosis of the infrarenal aorta).[18] Yet, it is important to note that with both nilotinib and ponatinib, arterial thromboembolism also occurred in young patients, without prior risk factor or without atherosclerotic lesion prior to TKI initiation.[6,16,22,23] For dasatinib and bosutinib, too low arterial occlusive events were reported to correlate risk factors and arterial occlusive events.[14] For the future, it will be interesting to obtain more data on these cardiovascular events that may occur during dasatinib and bosutinib treatments to identify patients at risk and develop predictive scores similarly than those existing with nilotinib.[24]

Method

Literature search was performed on PubMed on April 4, 2016, and updated on July 11, 2017. Articles published in languages other than English were excluded from the analysis. Primary criteria were pathophysiological explanation of arterial thrombotic events. Abstracts and full-text articles were reviewed with a focus on atherogenesis, plaque rupture, platelet functions, and their link with the development of arterial thrombosis with BCR-ABL TKIs. The reference section of identified articles was also examined.

Keywords Searched in Titles and Abstracts

- Vascular.
- Thrombosis.

Table S1 Clinical features of vascular occlusive events with new generations BCR-ABL TKIs

	Dasatinib	Nilotinib	Bosutinib	Ponatinib
Relative risk (OR; 95% CI)				
VOEs[25]	2.91 (1.43–5.94)	3.48 (2.21–5.49)	1.77 (0.54–5.83)	3.47 (1.23–9.78)
VTEs[26]	2.24 (0.68–7.42)	1.80 (0.45–7.15)	Unknown	7.29 (0.15–367.61)
AOEs[26]	3.32 (1.37–8.01)	3.69 (2.29–5.95)	1.77 (0.54–5.83)	3.26 (1.12–9.50)
Absolute risk (major AOEs) (/100 p-y)	1.1[27]	2.8[27]	Unknown	10.6[27]<br>First year: 15.1[14]
Arterial location	Coronary > PAOD > cerebral[2]<br>Coronary > cerebral > PAOD[28]	Coronary > cerebral > PAOD[2]<br>Coronary > PAOD > cerebral[5]<br>Coronary > cerebral = PAOD[3]	Unknown	Coronary > PAOD = cerebral[2,14,29]<br>Coronary > PAOD > cerebral[4,30]
Time to event (mo)[a]	19–22[17]	42–47[5,7]	Unknown	
Risk factors associated with VOE	Unknown	Age >65 y[5]<br>Dyslipidemia[7]<br>Gender: male[7]<br>High dose (800 mg)[3,7]<br>High homocysteinemia[7]<br>Cardiovascular risk factor at baseline[5]<br>High glycated hemoglobin[7]	Unknown	Age >60 y[14]<br>Prior VOE[14]<br>Arterial hypertension[14]<br>Diabetes[14]<br>High-dose ponatinib[14]

[a]Times to event for arterial occlusive disease.
Abbreviations: AOE, arterial occlusive event; CI, confidence interval; OR, odds ratio; PAOD, peripheral arterial occlusive disease; p-y, patient-year; VOE, vascular occlusive events; VTE, venous thromboembolism.

- Atherosclerosis.
- Arteriosclerosis.
- Cardiovascular.
- Venous.
- Arterial.
- Hemostasis.
- Metabolic.
- Metabolism.
- Glycemia.
- Glycaemia.
- Cholesterol.
- Triglyceride.
- Platelet.

Search *((((((((((((vascular[Title/Abstract]) OR thrombosis [Title/Abstract]) OR atherosclerosis[Title/Abstract]) OR arteriosclerosis[Title/Abstract]) OR cardiovascular[Title/Abstract]) OR venous[Title/Abstract]) OR arterial[Title/Abstract]) OR hemostasis[Title/Abstract]) OR metabolic[Title/Abstract]) OR metabolism[Title/Abstract]) OR glycemia[Title/Abstract]) OR glycaemia[Title/Abstract]) OR cholesterol[Title/Abstract]) OR triglyceride[Title/Abstract]) OR platelet[Title/Abstract]*

Imatinib

Search *(((imatinib[Title/Abstract]) OR imatinib mesylate [Title/Abstract]) OR STI-571[Title/Abstract]) OR STI571[Title/Abstract]*

Total: Search *(((((imatinib[Title/Abstract]) OR imatinib mesylate[Title/Abstract]) OR STI-571[Title/Abstract]) OR STI571[Title/Abstract])) AND ((((((((((((((((vascular[Title/Abstract]) OR thrombosis[Title/Abstract]) OR atherosclerosis[Title/Abstract]) OR arteriosclerosis[Title/Abstract]) OR cardiovascular[Title/Abstract]) OR venous[Title/Abstract]) OR arterial[Title/Abstract]) OR hemostasis[Title/Abstract]) OR metabolic[Title/Abstract]) OR metabolism[Title/Abstract]) OR glycemia[Title/Abstract]) OR glycaemia[Title/Abstract]) OR cholesterol[Title/Abstract]) OR triglyceride[Title/Abstract]) OR platelet[Title/Abstract]))))*

➔ **1,726 abstracts**.

Dasatinib

Search *((dasatinib[Title/Abstract]) OR BMS-354825[Title/Abstract]) OR BMS354825[Title/Abstract]*

Total: Search *((((dasatinib[Title/Abstract]) OR BMS-354825 [Title/Abstract]) OR BMS354825[Title/Abstract])) AND ((((((((((((((((vascular[Title/Abstract]) OR thrombosis[Title/Abstract]) OR atherosclerosis[Title/Abstract]) OR arteriosclerosis[Title/Abstract]) OR cardiovascular[Title/Abstract]) OR venous[Title/Abstract]) OR arterial[Title/Abstract]) OR hemostasis[Title/Abstract]) OR metabolic[Title/Abstract]) OR metabolism [Title/Abstract]) OR glycemia[Title/Abstract]) OR glycaemia[Title/Abstract]) OR cholesterol[Title/Abstract]) OR triglyceride[Title/Abstract]) OR platelet[Title/Abstract]))))*

➔ **247 abstracts**.

Nilotinib

Search *((nilotinib[Title/Abstract]) OR AMN107[Title/Abstract]) OR AMN-107[Title/Abstract]*

Total: Search *((((nilotinib[Title/Abstract]) OR AMN107[Title/Abstract]) OR AMN-107[Title/Abstract])) AND ((((((((((((((((vascular[Title/Abstract]) OR thrombosis[Title/Abstract]) OR atherosclerosis[Title/Abstract]) OR arteriosclerosis[Title/Abstract]) OR cardiovascular[Title/Abstract]) OR venous[Title/Abstract]) OR arterial[Title/Abstract]) OR hemostasis[Title/Abstract]) OR metabolic[Title/Abstract]) OR metabolism[Title/Abstract]) OR glycemia[Title/Abstract]) OR glycaemia[Title/Abstract]) OR cholesterol[Title/Abstract]) OR triglyceride[Title/Abstract]) OR platelet[Title/Abstract]))))*

➔ **193 abstracts.**

Bosutinib

Search *(((bosutinib[Title/Abstract]) OR SKI-606[Title/Abstract]) OR SKI606[Title/Abstract]*

Total: Search *((((bosutinib[Title/Abstract]) OR SKI-606[Title/Abstract]) OR SKI606[Title/Abstract])) AND ((((((((((((((((vascular[Title/Abstract]) OR thrombosis[Title/Abstract]) OR atherosclerosis[Title/Abstract]) OR arteriosclerosis[Title/Abstract]) OR cardiovascular[Title/Abstract]) OR venous[Title/Abstract]) OR arterial[Title/Abstract]) OR hemostasis[Title/Abstract]) OR metabolic[Title/Abstract]) OR metabolism[Title/Abstract]) OR glycemia[Title/Abstract]) OR glycaemia[Title/Abstract]) OR cholesterol[Title/Abstract]) OR triglyceride[Title/Abstract]) OR platelet[Title/Abstract]))))*

➔ **50 abstracts.**

Ponatinib

Search *(Ponatinib[Title/Abstract]) OR AP24534[Title/Abstract]*

Total: Search *(((Ponatinib[Title/Abstract]) OR AP24534 [Title/Abstract])) AND ((((((((((((((((vascular[Title/Abstract]) OR thrombosis[Title/Abstract]) OR atherosclerosis[Title/Abstract]) OR arteriosclerosis[Title/Abstract]) OR cardiovascular[Title/Abstract]) OR venous[Title/Abstract]) OR arterial [Title/Abstract]) OR hemostasis[Title/Abstract]) OR metabolic [Title/Abstract]) OR metabolism[Title/Abstract]) OR glycemia [Title/Abstract]) OR glycaemia[Title/Abstract]) OR cholesterol [Title/Abstract]) OR triglyceride[Title/Abstract]) OR platelet [Title/Abstract]))))*

➔ **66 abstracts.**

BCR-ABLTKIs

Total: Search *(BCR-ABL[Title/Abstract]) AND ((((((((((((((((vascular[Title/Abstract]) OR thrombosis[Title/Abstract]) OR atherosclerosis[Title/Abstract]) OR arteriosclerosis[Title/Abstract]) OR*

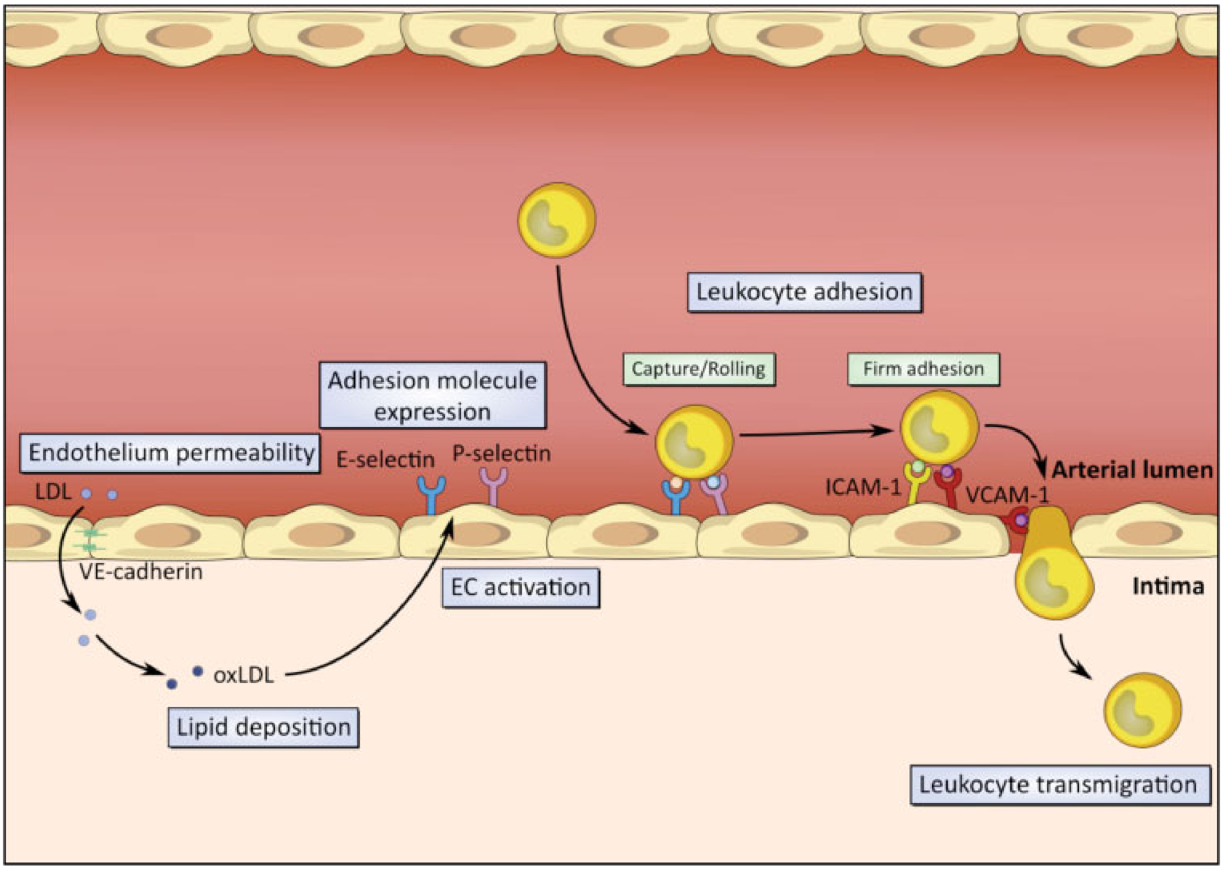

Fig. S1 Endothelial dysfunction initiates atherosclerosis. EC, endothelial cell; ICAM, intercellular adhesion molecule; LDL, low-density lipoprotein; oxLDL, oxidized low-density lipoprotein; TNF, tumor necrosis factor; VCAM, vascular cell adhesion molecule; VE-cadherin, vascular endothelial cadherin.

cardiovascular[Title/Abstract]) OR venous[Title/Abstract]) OR arterial[Title/Abstract]) OR hemostasis[Title/Abstract]) OR metabolic [Title/Abstract]) OR metabolism[Title/Abstract]) OR glycemia[Title/Abstract]) OR glycaemia[Title/Abstract]) OR cholesterol[Title/Abstract]) OR triglyceride[Title/Abstract]) OR platelet[Title/Abstract]))))

➔ **596 abstracts.**

Total: **2,878 abstracts.**

Without duplicates: **2,175 abstracts.**

A key event in the pathophysiology of atherosclerosis is the dysfunction of the endothelium, which results in endothelium permeability, enabling migration and trapping of lipoprotein into the intima. Modified lipoproteins in the intima activate endothelial cells and promotes the expression of cell adhesion molecules on the vessel wall, endorsing leukocyte migration into the intima.[31] Among adhesion molecules involved in atherosclerosis development, E-selectin, P-selectin, VCAM-1, and ICAM-1 possess major functions in leukocyte recruitment.[32] E-selectin and P-selectin are involved in the capture and rolling of monocytes, whereas ICAM-1 and VCAM-1 are implicated in leukocyte firm adhesion.[31] Monocyte transmigration is finally mediated by VCAM-1 and various chemokines produced by ECs, macrophages, and vascular smooth muscle cells.[32]

Additionally to the role of the endothelium in early atherosclerosis development, a recent in vivo study reports that vascular leakage is a marker to distinguish stable from rupture-prone atherosclerotic plaques (i.e., plaques are more prone to rupture subsequently to increase leukocytes migrating to the intima).[33]

Chronic inflammation is a key feature of atherosclerotic diseases and is involved in all stages of the disease, from atheroma formation to plaque rupture.[34] Inflammation is initiated by retention and oxidative modification of lipids and lipoproteins in the vessel wall that results in monocyte chemotaxis and recruitment of blood immune cells (particularly monocytes and T lymphocytes; ►**Fig. S1**).[34] Thereafter, immune cells initiate inflammatory reactions, with transformation of monocytes to macrophages and activation of T lymphocytes, predominantly to proinflammatory lymphocytes (i.e., Th1 subtype).[35] Macrophages within the atheroma uptake oxidized lipoprotein by scavenger receptor (e.g., SR-A and CD36) and transform to foam cells.[34] Macrophages also express and secrete matrix metalloproteinases (MMPs) that degrade extracellular matrix, thin the fibrous cap, and subsequently destabilize the atherosclerotic plaque.[36] Macrophages, foam cells, and activated T-cells release high content of proinflammatory mediators (e.g., IL-6, IL-12, and TNF- α), accentuating inflammation within the intima.

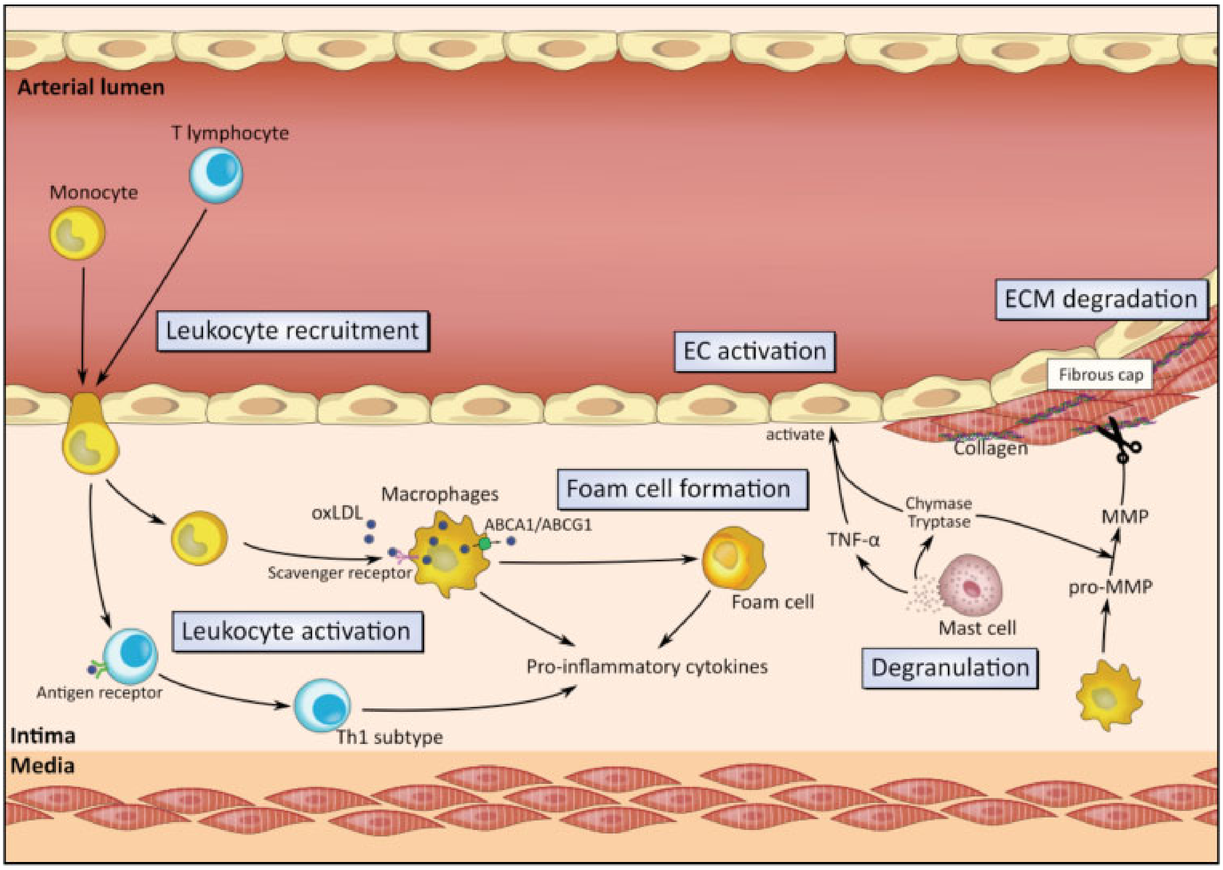

Fig. S2 Inflammation during atherosclerosis. ABC, ATP binding cassette; EC, endothelial cell; ECM, extracellular matrix; MMP, matrix metalloproteinase; oxLDL, oxidized low-density lipoprotein; TNF, tumor necrosis factor.

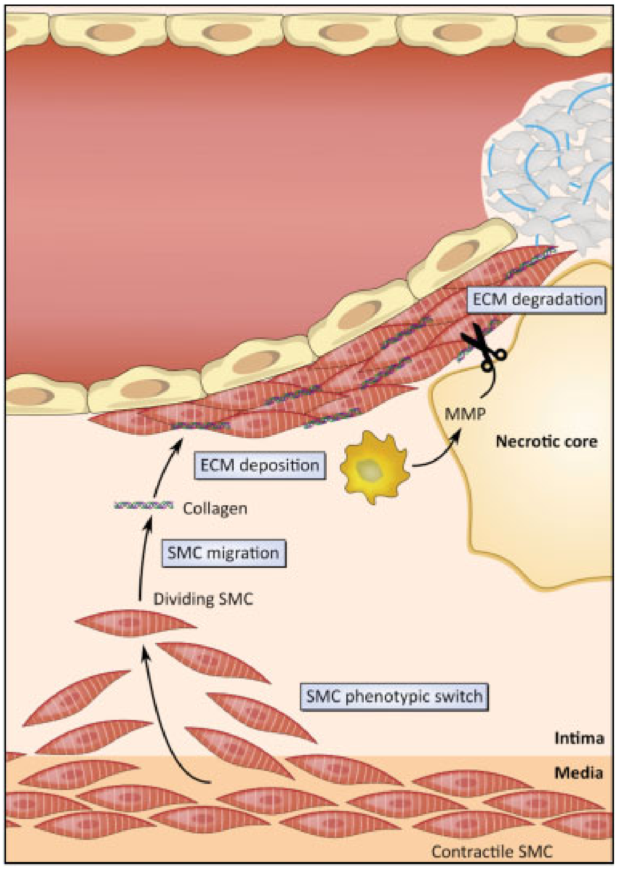

Fig. S3 Fibrous cap thickness and plaque rupture. ECM, extracellular matrix; MMP, matrix metalloproteinase; SMC, smooth muscle cell.

Finally, mast cells represent only a small proportion of cells in atherosclerosis but are important players in plaque growth and destabilization.[37] During atherosclerosis, they are recruited within the intima and may degranulate, inducing release of high content of TNF-α, tryptase and chymase, two enzymes able to activate MMP proform.[38] Episodes of acute inflammation are notably associated with rapid atherosclerosis development and play a role in plaque rupture or fissuring, increasing the inflammatory process within the plaque.[39]

Atherosclerosis is considered as a fibroproliferative disease. Indeed, during atherogenesis, vascular smooth muscle cells (VSMCs) undergo phenotypic switch from a contractile phenotype to a migratory and secretion phenotype. They acquire the capacity to migrate and produce extracellular matrix (ECM) proteins, additionally to a greater capacity to proliferate.[40] The stability of atherosclerotic plaque correlates to the thickness of its fibrous cap, which is composed of SMC in a collagenous-proteoglycan matrix. Different mechanisms may contribute to fibrous cap thickening, notably a high MMP concentration (►**Fig. S2**) or high SMC mortality that directly reduces ECM content in the fibrous cap. Therefore, the content of VSMCs within the plaque and their capacity to secrete ECM components directly correlate with the thickness of the fibrous cap and therefore play a major role to differentiate rupture-prone plaque from stable plaque.[40] Additionally, VSMCs are involved in plaque repair in case of rupture through their capacity to proliferate and synthesize ECM. Thus, the presence of VSMCs in advanced lesions is beneficial and the

Table S2 Inhibitory profiles of BCR-ABL TKIs on tyrosine kinase

	Imatinib		Dasatinib		Nilotinib		Bosutinib		Ponatinib	
Lmr1										
Lmr2										
Lmr3										
Tyk2	O	42,43	X[a]	42,43,48,50,52	O	43,50	O	42,50,52		
JAK1	O	41,42,46	O	41,42	X[a]	41,46	O	41,42	X	41
JAK2	O	44,45			O	44			X	53
JAK3										
Ack	O	42	X	42,48			X	42		
Tnk1	O	42	O	42			O	42		
HER3										
EGFR	O	53	X	48,51,52	O	53	X	52	O	53
HER2	O	53			O	49,53			O	53
HER4			X	52			X	52		
TIE1										
TIE2 = Tek	O	45,46			X	46,49				
Ret	O	41,44	X[a]	41,48	O	41,44	O	41,53	X	41,53
FGFR1	O	42,44	O	42	O	44	X*	42,52		
FGFR2	O	41	X[a]	41,52	O	41	O	41,52	X	41

(*Continued*)

Table S2 (*Continued*)

	Imatinib		Dasatinib		Nilotinib		Bosutinib		Ponatinib	
FGFR3	O	41	O	41	O	41	O	41	X	41
FGFR4										
FLT1 = VEGFR1	O	41,45	O	41,52	O	41	X[a]	41,52	X	41
KDR = VEGFR2	O	41,44,45	X[a]	41,50	O	41,49,50	O	41,50	X	41
FLT4 = VEGFR3	O	41	O	41	O	41	O	41	X	41
Fms/CSFR	X[a]	41,45	X	41,52	X	41,49	X[a]	41,52	X	41
Kit	X[a]	41,42,44,45,48	X[a]	41,42,48,53	X	41,44,48,49	O	41,42	X	41,53
FLT3	O	41,44,45	X[a]	41,50	O	41,44,50	O	41,50	X	41,53
PDGFRα	X	41,44,45,48	X[a]	41,48,52	X	41,44,48,49	O	41,52	X	41
PDGFRβ	X	41,44,45,48	X	41,48,50,52	X[a]	41,44,48–50	O	41,50,52	X	41
CCK4/PTK7										
LTK										
ALK	O	42	O	42			X	42		
Ros										
InsR	O	44			O	44				
IGF1R	O	44			O	44				
IRR										
DDR1	X[a]	42,43,47,48	X[a]	42,43,48	X	43,48,49	X[a]	42,52	X	47
DDR2	X	41,47	X	41,48,52	X[a]	41,49	X	41,52	X	41,47
MuSK										
TRKA	O	42	X[a]	42,52			X[a]	42,52		
TRKB	O	41	X[a]	41,52	O	41	X[a]	41,52	X	41
TRKC	O	41	O	41	O	41	O	41	O	41
ROR1	X	41	X	41	X	41	O	41	X	41
ROR2										
Ron										
Met	O	41,44,45	O	41	O	41,44	O	41	O	41,53
Axl	O	41	O	41,52	O	41	X[a]	41,52	O	41
Mer	O	41,42	O	41,42	O	41	X[a]	41,42	O	41
Tyro3/Sky	O	41	O	41,52	O	41	X[a]	41,52	O	41
RYK										
Fer	O	42	O	42,50	O	50	X	42,50,52		
Fes			O	50	O	50	X	50,52		
Abl	X	41,43,48	X	41,43,48,50,52	X	41,43,48,50	X	41,50,52	X	41,53
Arg	X	41,42,48	X	41,42,48,50,52	X[a]	41,44,48–50	X	41,42,50,52	X	41,53
CSK	O	42,43	X	42,43,48,50,51	X[a]	43,49,50	X	42,50,52		
CTK										
Etk/BMX	O	41,43	X	41,43,48,52	O	41,43	X	41,52	X	41
BTK	O	42,43	X	42,43,48,50,51	X[a]	43,50	X	42,50		
TXK			X	48,52			X	52		
TEC	O	42,43	X	42,43,48,50,52	O	43,50	X[a]	42,50,52		

Table S2 (*Continued*)

	Imatinib		Dasatinib		Nilotinib		Bosutinib		Ponatinib	
ITK										
Brk			X	48,52			X	52		
Srm										
FRK	O	41–43	X[a]	41–43,48,50,52	X[a]	41,43,49,50	X	41,42,50,52	X	41
BLK	O	41	X	41,48,50,52	X[a]	41,49,50	X	41,50,52	X	41
Lck	O	41,43	X	41,43,48,50,52	X[a]	41,43,49,50	X	41,50,52	X	41
HCK	O	42,43	X	42,43,48,50,51	X[a]	43,49,50	X	42,50	X	41
Lyn	O	41–43,53	X	41–43,48,50,51	X[a]	41,43,49,50	X	41,42,50	X	41,53
Fgr	O	41,43	X	41,43,48,50,52	O	41,43,49	X	41,52	X	41
Fyn	O	41–43	X	41–43,48,50,51	X[a]	41,43,49,50	X	41,42,50	X	41
Src	O	41–43,45	X	41–43,48,50,51	O	41,43,49,50	X	41,42,50	X	41
Yes	O	43	X	43,48,50–52	O	43,49,50	X	50,52	X	53
EphA1			X	52	X	49	X	52		
EphA2			X	48,52	X	49	X	52		
EphA3	O	41	X	41,48,52	X[a]	41,49	X	41,52	X	41
EphA4	O	41	X	41,48,52	X	41,49	X	41,52	X	41
EphA5	O	41	X	41,48,52	X	41	X	41,52	X	41
EphA6										
EphA7										
EphA8			X	48,52	X	49	X	52		
EphA10										
EphB1	O	41,42	X[a]	41,42,48,52	X	41	X	41,42,52	X	41
EphB2	O	41–43,46	X	41–43,48,52	X	41,43,46	X[a]	41,42,52	X	41
EphB3			X	50,52	O	50	X	50,52		
EphB4	O	41–43	X	41–43,48,50,52	X[a]	41,43,49,50	X	41,42,50,52	X	41,53
EphB6	O	43	X	43,48,52	X[a]	43,49				
FAK	O	42	X[a]	42,48,50	O	50	X	42,50		
PYK2	O	42	O	42,50	X	50	X	42,50		
Syk	O	42,43	X	42,43,48,50	O	43	X	42		
ZAP70										
JAK1~b										
Tyk2~b										
JAK2~b										
JAK3~b										
SuRTK106										

Abbreviation: TKI, tyrosine kinase inhibitor.
Notes: Gray boxes indicate unknown effect of BCR-ABL TKIs on the tyrosine kinase. Red and green boxes indicate, respectively, no inhibition and inhibition of the tyrosine kinase by the TKI. Second columns indicate references. Similarly, numbers in red indicate no inhibition of the tyrosine kinase by the TKI, whereas numbers in green indicate inhibition.
[a]Discordant results between the experiments.

destabilization of atherosclerotic plaques might be due to alteration of the balance between VSMC proliferation and migration versus VSMC death and senescence that might promote atherogenesis and plaque instability.[40]

References

1 Dahlén T, Edgren G, Lambe M, et al; Swedish CML Group and the Swedish CML Register Group. Cardiovascular events associated with use of tyrosine kinase inhibitors in chronic myeloid leukemia: a population-based cohort study. Ann Intern Med 2016;165 (03):161–166

2 Sam PY, Ahaneku H, Nogueras-Gonzalez GM, et al. Cardiovascular events among patients with chronic myeloid leukemia (CML) treated with tyrosine kinase inhibitors (TKIs). Blood 2016;128(22):1919

3 Hochhaus A, Saglio G, Hughes TP, et al. Long-term benefits and risks of frontline nilotinib vs imatinib for chronic myeloid leukemia in chronic phase: 5-year update of the randomized ENESTnd trial. Leukemia 2016;30(05):1044–1054

4 Mauro MJ, Cortes JE, Kantarjian HM, et al. Ponatinib in chronic-phase chronic myeloid leukemia patients: final report from a phase 1 trial. Blood 2016;128(22):3063

5 Gugliotta G, Castagnetti F, Breccia M, et al; GIMEMA CML Working Party. Long-term outcome of a phase 2 trial with nilotinib 400. mg twice daily in first-line treatment of chronic myeloid leukemia. Haematologica 2015;100(09):1146–1150

6 Stève-Dumont M, Baldin B, Legros L, et al. Are nilotinib-associated vascular adverse events an under-estimated problem? Fundam Clin Pharmacol 2015;29(02):204–208

7 Fossard G, Blond E, Balsat M, et al. Hyperhomocysteinemia and high doses of nilotinib favor cardiovascular events in chronic phase chronic myelogenous leukemia patients. Haematologica 2016;101(03):e86–e90

8 Lyaker MR, Tulman DB, Dimitrova GT, Pin RH, Papadimos TJ. Arterial embolism. Int J Crit Illn Inj Sci 2013;3(01):77–87

9 European Medicines Agency. Tasigna - Summary of Product Characteristics 2017. Available at: http://www.ema.europa.eu/docs/en_GB/document_library/EPAR_-_Product_Information/human/000798/WC500034394.pdf. Accessed December 22, 2017

10 European Medicines Agency. Iclusig - Summary of Product Characteristics 2017. Available at: http://www.ema.europa.eu/docs/en_GB/document_library/EPAR_-_Product_Information/human/002695/WC500145646.pdf. Accessed December 22, 2017

11 European Medicines Agency. Glivec - Summary of Product Characteristics 2017. Available at: http://www.ema.europa.eu/docs/en_GB/document_library/EPAR_-_Product_Information/human/000406/WC500022207.pdf. Accessed December 22, 2017

12 Kim TD, Rea D, Schwarz M, et al. Peripheral artery occlusive disease in chronic phase chronic myeloid leukemia patients treated with nilotinib or imatinib. Leukemia 2013;27(06):1316–1321

13 Aichberger KJ, Herndlhofer S, Schernthaner GH, et al. Progressive peripheral arterial occlusive disease and other vascular events during nilotinib therapy in CML. Am J Hematol 2011;86(07):533–539

14 Cortes JE, Kim D-W, Pinilla-Ibarz J, et al. Long-term follow-up of Ponatinib efficacy and safety in the phase 2 PACE trial. Blood 2014;124(21):3135

15 Cortes J, Pinilla-Ibarz J, le Coutre P, et al. 4-year results from the Pivotal Phase 2 PACE Trial: efficacy and safety in heavily pretreated leukemia patients. Clin Lymphoma Myeloma Leuk 2016;16:S56

16 Bondon-Guitton E, Combret S, Pérault-Pochat MC, et al. Cardiovascular risk profile of patients with peripheral arterial occlusive disease during nilotinib therapy. Target Oncol 2016;11(04):549–552

17 Gora-Tybor J, Medras E, Calbecka M, et al. Real-life comparison of severe vascular events and other non-hematological complications in patients with chronic myeloid leukemia undergoing second-line nilotinib or dasatinib treatment. Leuk Lymphoma 2015;56(08):2309–2314

18 Brauchli YB, Wais T, Gratwohl A, et al. Fatal myocardial infarction during nilotinib treatment in a 60-year-old male patient. Acta Oncol 2010;49(04):523–525

19 Maurizot A, Beressi JP, Manéglier B, et al. Rapid clinical improvement of peripheral artery occlusive disease symptoms after nilotinib discontinuation despite persisting vascular occlusion. Blood Cancer J 2014;4:e247

20 Gautier V, Mirault T, Azarine A, et al. [Peripheral artery occlusive disease of the lower limbs: Rapid aggravation in a patient taking nilotinib for chronic myeloid leukemia]. J Mal Vasc 2015;40(04):231–239

21 Tefferi A. Nilotinib treatment-associated accelerated atherosclerosis: when is the risk justified? Leukemia 2013;27(09):1939–1940

22 Tefferi A, Letendre L. Nilotinib treatment-associated peripheral artery disease and sudden death: yet another reason to stick to imatinib as front-line therapy for chronic myelogenous leukemia. Am J Hematol 2011;86(07):610–611

23 Mirault T, Rea D, Azarine A, Messas E. Rapid onset of peripheral artery disease in a chronic myeloid leukemia patient without prior arterial disorder: direct relationship with nilotinib exposure and clinical outcome. Eur J Haematol 2015;94(04):363–367

24 Breccia M, Molica M, Zacheo I, Serrao A, Alimena G. Application of systematic coronary risk evaluation chart to identify chronic myeloid leukemia patients at risk of cardiovascular diseases during nilotinib treatment. Ann Hematol 2015;94(03):393–397

25 Douxfils J, Haguet H, Mullier F, Chatelain C, Graux C, Dogné JM. Association between BCR-ABL tyrosine kinase inhibitors for chronic myeloid leukemia and cardiovascular events, major molecular response, and overall survival: a systematic review and meta-analysis. JAMA Oncol 2016

26 Haguet H, Douxfils J, Mullier F, Chatelain C, Graux C, Dogné JM. Risk of arterial and venous occlusive events in chronic myeloid leukemia patients treated with new generation BCR-ABL tyrosine kinase inhibitors: a systematic review and meta-analysis. Expert Opin Drug Saf 2017;16(01):5–12

27 Chai-Adisaksopha C, Lam W, Hillis C. Major arterial events in patients with chronic myeloid leukemia treated with tyrosine kinase inhibitors: a meta-analysis. Leuk Lymphoma 2016;57(06):1300–1310

28 Cortes JE, Saglio G, Kantarjian HM, et al. Final 5-year study results of DASISION: the Dasatinib versus Imatinib study in treatment-naïve chronic myeloid leukemia patients trial. J Clin Oncol 2016;34(20):2333–2340

29 Lipton JH, Chuah C, Guerci-Bresler A, et al; EPIC investigators. Ponatinib versus imatinib for newly diagnosed chronic myeloid leukaemia: an international, randomised, open-label, phase 3 trial. Lancet Oncol 2016;17(05):612–621

30 Jabbour EJ, Cortes JE, Talpaz M, et al. Long-term follow-up of the efficacy and safety of Ponatinib in Philadelphia chromosome-positive leukemia patients with the T315I mutation. Blood 2016;128(22):3067

31 Mestas J, Ley K. Monocyte-endothelial cell interactions in the development of atherosclerosis. Trends Cardiovasc Med 2008;18 (06):228–232

32 Moore KJ, Sheedy FJ, Fisher EA. Macrophages in atherosclerosis: a dynamic balance. Nat Rev Immunol 2013;13(10):709–721

33 Phinikaridou A, Andia ME, Lavin B, Smith A, Saha P, Botnar RM. Increased vascular permeability measured with an albumin-binding magnetic resonance contrast agent is a surrogate marker of rupture-prone atherosclerotic plaque. Circ Cardiovasc Imaging 2016;9(12):e004910

34 Manduteanu I, Simionescu M. Inflammation in atherosclerosis: a cause or a result of vascular disorders? J Cell Mol Med 2012;16 (09):1978–1990

35 Gorbet MB, Sefton MV. Biomaterial-associated thrombosis: roles of coagulation factors, complement, platelets and leukocytes. Biomaterials 2004;25(26):5681–5703

36 Silvestre-Roig C, de Winther MP, Weber C, Daemen MJ, Lutgens E, Soehnlein O. Atherosclerotic plaque destabilization: mechanisms, models, and therapeutic strategies. Circ Res 2014;114(01):214–226

37 Shi GP, Bot I, Kovanen PT. Mast cells in human and experimental cardiometabolic diseases. Nat Rev Cardiol 2015;12(11):643–658

38 Libby P. Inflammation in atherosclerosis. Nature 2002;420(6917):868–874

39 Jackson SP. Arterial thrombosis–insidious, unpredictable and deadly. Nat Med 2011;17(11):1423–1436

40 Bennett MR, Sinha S, Owens GK. Vascular smooth muscle cells in atherosclerosis. Circ Res 2016;118(04):692–702

41 Rivera VM, Pritchard JR, Gonzalvez F, Baker T, Gozgit JM, Hodgson G. Comparative TKI profiling analyses to explore potential mechanisms of Ponatinib-associated arterial thrombotic events. Blood 2014;124(21):1783

42 Bantscheff M, Eberhard D, Abraham Y, et al. Quantitative chemical proteomics reveals mechanisms of action of clinical ABL kinase inhibitors. Nat Biotechnol 2007;25(09):1035–1044

43 Rix U, Hantschel O, Dürnberger G, et al. Chemical proteomic profiles of the BCR-ABL inhibitors imatinib, nilotinib, and dasatinib reveal novel kinase and nonkinase targets. Blood 2007;110(12):4055–4063

44 Weisberg E, Manley PW, Breitenstein W, et al. Characterization of AMN107, a selective inhibitor of native and mutant Bcr-Abl. Cancer Cell 2005;7(02):129–141

45 Buchdunger E, Cioffi CL, Law N, et al. Abl protein-tyrosine kinase inhibitor STI571 inhibits in vitro signal transduction mediated by c-kit and platelet-derived growth factor receptors. J Pharmacol Exp Ther 2000;295(01):139–145

46 Albrecht-Schgoer K, Huber K, Grebien F, et al. Nilotinib exerts direct pro-atherogenic and anti-angiogenic effects on vascular endothelial cells: a potential explanation for drug-induced vasculopathy in CML. Blood 2013;122(21):257

47 Canning P, Tan L, Chu K, Lee SW, Gray NS, Bullock AN. Structural mechanisms determining inhibition of the collagen receptor DDR1 by selective and multi-targeted type II kinase inhibitors. J Mol Biol 2014;426(13):2457–2470

48 Hantschel O, Rix U, Superti-Furga G. Target spectrum of the BCR-ABL inhibitors imatinib, nilotinib and dasatinib. Leuk Lymphoma 2008;49(04):615–619

49 Manley PW, Drueckes P, Fendrich G, et al. Extended kinase profile and properties of the protein kinase inhibitor nilotinib. Biochim Biophys Acta 2010;1804(03):445–453

50 Fabian MA, Biggs WH III, Treiber DK, et al. A small molecule-kinase interaction map for clinical kinase inhibitors. Nat Biotechnol 2005;23(03):329–336

51 Shi H, Zhang CJ, Chen GY, Yao SQ. Cell-based proteome profiling of potential dasatinib targets by use of affinity-based probes. J Am Chem Soc 2012;134(06):3001–3014

52 Remsing Rix LL, Rix U, Colinge J, et al. Global target profile of the kinase inhibitor bosutinib in primary chronic myeloid leukemia cells. Leukemia 2009;23(03):477–485

53 Green MR, Newton MD, Fancher KM. Off-Target Effects of BCR-ABL and JAK2 Inhibitors. Am J Clin Oncol 2016;39(01):76–84
